# Life history and nesting ecology of a Japanese tube-nesting spider wasp *Dipogon sperconsus* (Hymenoptera: Pompilidae)

**DOI:** 10.1038/s41598-021-92124-z

**Published:** 2021-06-17

**Authors:** Yutaka Nishimoto, Akira Shimizu, Jin Yoshimura, Tomoji Endo

**Affiliations:** 1 Takarazuka, Hyogo 669-1211 Japan; 2 Department of Biological Science, Tokyo Metropolitan University, Hachioji, Tokyo 192-0397 Japan; 3 Research Institute of Evolutionary Biology, Inc., Setagaya-ku, Tokyo 158-0098 Japan; 4 University Museum, The University of Tokyo, Bunkyo-ku, Tokyo 113-0033 Japan; 5 Department of International Health and Medical Anthropology, Institute of Tropical Medicine, Nagasaki University, Nagasaki, 852-8523 Japan; 6 Marine Biosystems Research Center, Chiba University, Uchiura, Kamogawa, Chiba 299-5502 Japan; 7 Department of Biosphere Sciences, School of Human Sciences, Kobe College, Nishinomiya, Hyogo 662-8505 Japan

**Keywords:** Evolution, Ecology, Behavioural ecology, Ecophysiology, Zoology, Entomology

## Abstract

To clarify the life history of the Japanese spider wasp *Dipogon sperconsus*, bionomical studies using bamboo-cane trap nests were carried out in Japan. Based on weekly and consecutive daily surveys of trap nests and rearing of broods from collected nests, we evaluated the production of cells and eggs per day, prey spiders, and seasonal patterns of nesting activities. We found a relatively short critical period of switching from the summer generation into the overwintering generation. We also found that the voltinism is affected by the timing of egg production of the second generation in relation to this critical period. The developmental period for each generation and sex, voltinism and cell production per day were determined based on data for a large number of individuals for the first time.

## Introduction

The family Pompilidae is one of the large groups of stinging wasps (Aculeata), members of which are well known as spider hunting wasps. A majority of the species attack spiders, sting them into paralysis and transport them to their nest-cells prepared before or after hunting. There are various modes of nest-provisioning: burrowing, building and renting^1^. Some species utilize pre-existing cavities as their nests. Among them, *Dipogon* Fox and *Auplopus* Spinola (subfamily Pepsinae) are the two large representative groups of tube-nesting species. In fact, almost all pompilid wasps emerging from bamboo cane trap nests belong to the above two genera^2,3^.

The female of the subgenus *Deuteragenia* Sustera of the genus *Dipogon* prepares a nest-cell before hunting (Recently the subgenus was elevated to a genus based on morphology-based cladistic analysis by Lelej & Loktinov^4^). After that, she carries the spider into the cell, grasping it by the spinnerets and walking sideway. In the cell, she places the spider on its venter and lays an egg on its abdomen anterolaterally. To make cell partitions and a closing plug, she carries clods of mud, sand grains, pieces of dead leaves, pebbles and other materials held in a fascicle of long curved bristles arising from the maxillary cardo (so-called ‘beard’)^5^. Then, she packed these materials with the dorsal tip of her gaster. Finally, a series of partitioned cells are constructed within a nest, and each cell contains a single cocoon. In Japan, a few papers on the nesting behavior and prey spiders of *Dipogon* (*Deuteragenia*) have been published^6,7^, but its detailed life history remains unknown. Outside of Japan, there are only a few records of the period from egg to emergence and voltinism of *Deuteragenia* (e.g., *Dipogon sayi sayi* Banks^2,8,9^ and *D. papago anomalus* Dreisbach^2^). Taxonomic studies on the Japanese species of *Dipogon* have been accomplished and 21 species of the genus have been reported from Japan^5,10–14^.

Trap nests have been widely used as a method for studies of the natural history, trophic interactions, biodiversity and applied ecology of cavity-nesting wasps and bees in vast areas^2,8,9,15–20^. In Japan, the trap-nest technique using bamboo canes has been adopted to clarify the nesting behaviors and life histories of these wasps and bees^1,3,21–23^. In Hyogo Prefecture, such studies were carried out in the Muko-gawa River and Chikusa***-***gawa River basins^24^. According to these studies, more than 28 species of wasps and bees were recorded in bamboo cane nests. Of them, six species were spider wasps belonging to the genus *Dipogon* (Pompilidae, Pepsinae), and the proportion of *Dipogon* nests to all nests was 18.2%. Similar investigations in southern Gifu Pref., Honshu^25,26^ and western Kochi Pref., Shikoku (Nishimoto et al*.*, unpublished data) showed that 19 and 17 species of tube-nesting wasps and bees were obtained, six and three of which belonged to *Dipogon*, and the proportion of their nests to all nests was 17.7 and 10.6%, respectively. Moreover, a similar investigation in a forest in western Tokyo showed that 6.9% of all trap nests were used by 19 species of wasps and bees, four of which belonged to *Dipogon*^3^. It was thus found that this genus is one of the predominant taxa in trap nest investigations in woody environments. Investigation of the fundamental bionomics and differences by species in the genus is important for the estimation of insect biodiversity in forests.

In this paper, we report the life history and nesting ecology of *Dipogon* (*Deuteragenia*) *sperconsus* Shimizu & Ishikawa, which is distributed in Japan from Hokkaido to Kyushu^5^ and is one of the most dominant Japanese species of *Dipogon*. This species constructs a consecutive series of cells in pre-existing cavities of dead trees (‘renting type of nesting’ after Iwata^1^). We can easily collect wasps of this species by using the trap-nest technique, in which bamboo-cane trap nests are set up in early spring. In past studies, the cane nests were returned to the laboratory in late autumn or winter when wasps in the nests were at their prepupal stage. Consequently, we obtained only knowledge concerning the overwintering generation, and thus the life history throughout the year remained unknown. In the present study, we elucidated the life history of this species in detail by using the trap-nest technique combined with weekly and consecutive daily surveys of the trap nests all year around and rearing of the broods in the nests. The developmental periods for each generation and sex, voltinism, and cell productivity per day are reported based on the collected data for a large number of individuals for the first time. To the best of our knowledge, this is the first estimation of the life history of a spider wasp, because such estimation is only possible with the daily consecutive surveys over the entire summer season.

## Results and discussion

### Nesting records

*Dipogon* nests were created singly per cane, because there were no examples in which wasps of two species emerged from the same cane in the study site. Thus, we designate “utilized canes” as “nests”.

**Figure 1:**
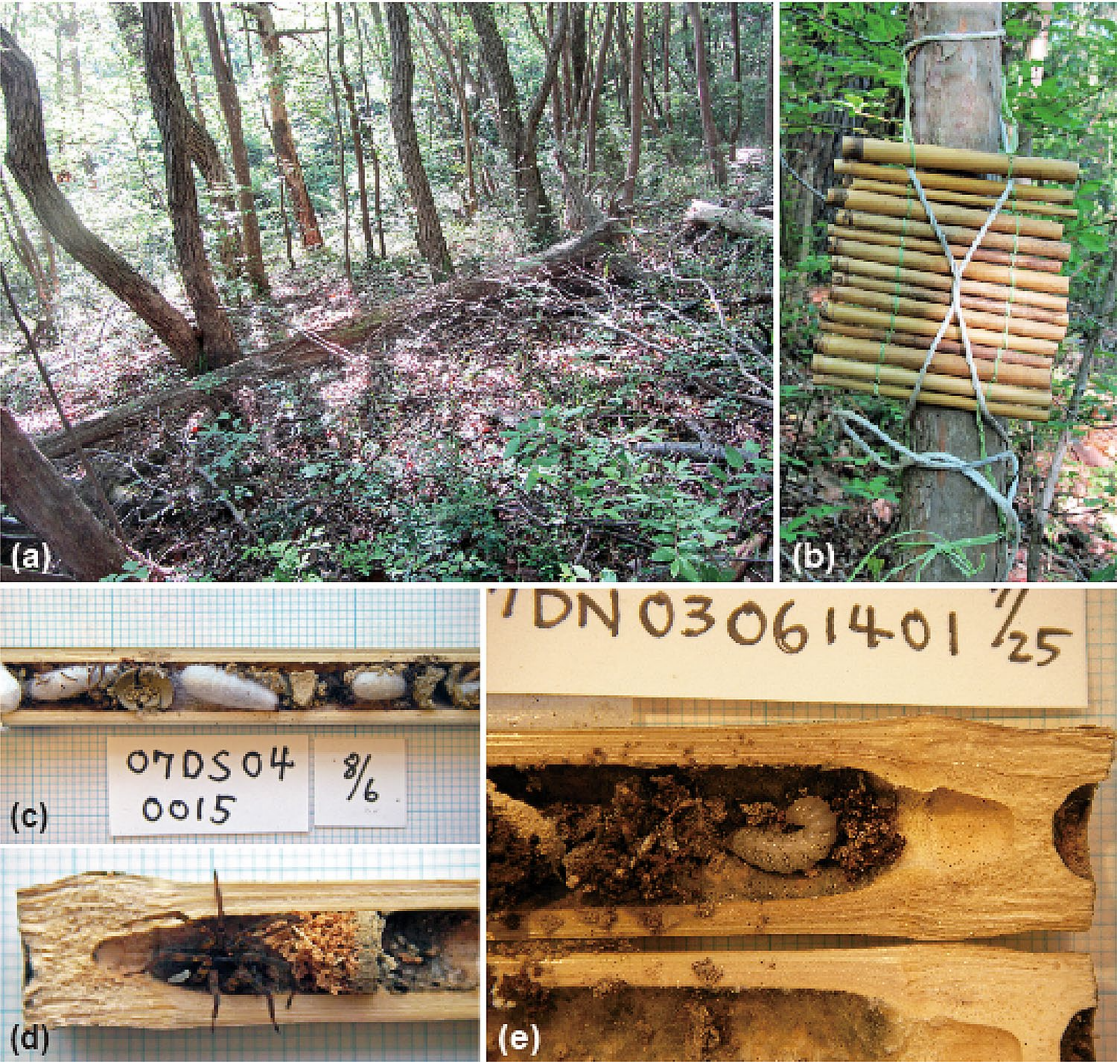
The study site in Kirihata, Takarazuka City, Hyogo Pref., Japan, and trap nests. (**a**) An old pine forest in which trap nests were installed. (**b**) A set of trap nests (cane bundle), 15 mixed-size bamboo canes bound vertically with vinyl-covered wires like a screen, attached to a tree trunk approximately 1.5 m above the ground. (**c**) A nest of *D. sperconsus*; this cane was installed in Shibutani, Takarazuka, Hyoto Pref. about 1 km southeast of the present study site on 29 July 2007 and was withdrawn on 6 August 2007. (**d**) A nest (6–5-5–1) of *D. sperconsus*; this cane was installed in Kirihata, Takarazuka, Hyoto Pref. about 500 m west-southwest of the present study site on 25 August 2010 and was withdrawn on 27 August 2010 (prey spider, *Agelena limbata* Thorell). (**e**) A nest of *D. sperconsus*; this cane was installed in Najio, Nishinomiya, Hyoto Pref. about 10 km southwest of the present study site on 15 July 2007 and was withdrawn on 25 July 2007. The minimum grid in the background graph paper of (**c**)–(**e**) is 1 mm. All photos taken by Y. Nishimoto.

In the four years, in pine forests in Takarazuka, Hyogo, Japan, we collected a total of 419 nests with 1033 cells from which species of *Dipogon* emerged (Fig. 1; Table 1; Supplementary Table S1). The numbers of nests and cells and the average and SD of the number of cells per nest for each species are shown in Table 1. Other wasps, bees, and parasitic wasps and flies also emerged from our trap nests (Supplementary Table S2), but we did not consider their nesting in the following analyses. Among 1033 cells, *D. sperconsus* emerged from 623 cells, *D. inconspersus* from 26 cells, and *D. bifasciatus* (Geoffroy) from 4 cells, while rearing failure occurred in 380 cells (Table 1), the owners of which we designate as “unknown *Dipogon* spp.” Based on the total cells of *Dipogon*, the proportion of cells constructed by *D. sperconsus* was 60.3% (623/1033*100), that of *D. inconspersus* was 2.5% (26/1033*100), and that of *D. bifasciatus* was 0.39% (4/1033*100). Based on the cells of the identified species, the proportion of cells constructed by *D. sperconsus* was 95.4% (623/(623 + 26 + 4)*100), that of *D. inconspersus* was 4.0% (26/(623 + 26 + 4)*100), and that of *D. bifasciatus* was 0.6% (4/(623 + 26 + 4)*100). From these proportions, we can estimate the number of cells constructed by the three species of *Dipogon* in the total 1033 *Dipogon* cells as ca. 985.5 cells (1033*0.954) by *D. sperconsus*, ca. 41.3 cells (1033*0.04) by *D inconspersus*, and ca. 6.2 cells (1033*0.006) by *D. bifasciatus*.

**Table 1:** The numbers of the collected nests and brood cells, and the mean number of cells per nest in three species of *Dipogon* (*Deuteragenia*).

Species/Year	2013	2014	2015	2016	Total
No. of bamboo canes installed	480	480	1050	1035	3045
*Dipogon sperconsus*					
No. cells/No. nests	131/43	84/38	168/64	240/76	623/221
Mean no. cells per nest ± SD	3.05 ± 1.81	2.21 ± 1.07	2.63 ± 1.20	3.16 ± 1.96	2.82 ± 1.63
*D. inconspersus*					
No. cells/No. nests	15/4	0/0	0/0	11/3	26/7
Mean no. cells per nest ± SD	3.75 ± 2.22	–	–	3.67 ± 1.15	3.71 ± 1.70
*D. bifasciatus*					
No. cells/No. nests	0/0	0/0	0/0	4/1	4/1
Mean no. cells per nest ± SD	–	–	–	4.00	4.00
Unknown					
No. cells/No. nests	107/52	43/25	165/80	65/33	380/190
Mean no. cells per nest ± SD	2.06 ± 1.04	1.72 ± 0.74	2.06 ± 1.09	1.97 ± 0.95	2.00 ± 1.01
Total (No. cells/No. nests)					1033/419

Because multiple cells were often constructed in a single nest, the number of nests was much smaller than the number of constructed cells. Among the 419 nests, 221 nests belonged to *D. sperconsus*, 7 nests belonged to *D. inconspersus*, and a single nest belonged to *D. bifasciatus*, but the remaining 190 nests could not be identified because of rearing failure (Table 1). The proportions of the nests in the three *Dipogon* species were calculated as follows: 96.5% (221/(221 + 7 + 1)*100) in *D. sperconsus*, 3.1% (7/(221 + 7 + 1)*100) in *D inconspersus*, and 0.4% (1/(221 + 7 + 1)*100) in *D. bifasciatus*. Thus, the estimated number of nests in each species was ca. 404.3 (419*0.965) in *D. sperconsus*, ca. 13.0 (419*0.031) in *D inconspersus*, and ca. 1.7 (419*0.004) in *D. bifasciatus*.

Next, we considered whether the cane bundles were used randomly. Based on the yearly frequency distributions of nests (Supplementary Tables S3–S6), we developed a null hypothesis assuming the nests are randomly distributed over bundles, where a negative binomial distribution is expected (Supplementary Tables S7–S8). Our yearly data indicate that the null hypothesis was rejected and that nests were more or less aggregated in a few bundles (Supplementary Figure S1; test statistics, Supplementary Table S8). This aggregation tendency (e.g., no nests in some bundles) may imply that some selected sites for bundles are not appropriate for *D. sperconsus*, for some unknown behavioral reasons. Further studies are needed to verify the habitat use of this species.

Yearly frequency distributions of the number of cells show that the range of cells constructed by *D. sperconsus* and unknown *D*. spp. combined were 1–10 cells, and the median was 2 cells (Supplementary Table S3–S6, Supplementary Figure S2). Most of the nests included 1–3 cells, and five or more cells were very rare. Most of the nests with many cells (e.g., 7–10 cells) were likely to be constructed by a single wasp because these wasps avoid interactions with other spider wasps. The average number of *D. sperconsus* cells per nest was 2.82 for four years, varying from 2.21 (2014) to 3.16 (2016) (Table 1), and the yearly differences were significant (Kruskal–Wallis test, $\chi ^{2} = 7.70$, *df* = 3, *p* = 0.05). In contrast, the average number of cells per nest of *D. sayi sayi* was slightly greater than that of *D. sperconsus*: 3.2 (1–6, SD = 1.47, n = 41) in the first generation and 4.7 (1–13, SD = 2.52, n = 107) in the second generation in Wisconsin, USA^8^; and 6.2 (1961), 4.0 (1962) and 3.0 (1963) in the summer generation and 7.5 (1961) and 3.2 (1962) in the overwintering generation in Northwestern Ontario^9^.

### Life history of Dipogon sperconsus

#### Developmental period

The developmental period of reared wasps was estimated in the summer and overwintering generations separately (Table 2, Supplementary Figure S3, Supplementary Tables S9–S12). In the summer generation, both females and males developed from egg to adult over approximately three weeks (23.1 days for females and 21.6 days for males; Table 2). There was no significant difference between sexes (*t*-test, after adjustment by Bonferroni method: *p* > 0.05). In the overwintering generation, approximately eight months were required from egg to adult (246 days for females and 247 days for males). There was also no significant difference between sexes (*t*-test, after adjustment by Bonferroni method: *p* > 0.05). In females, all developmental periods were significantly longer in the overwintering generation than in the summer generation (*t*-test, after adjustment by Bonferroni method: *p* < 0.05). In males, a similar tendency was found, but there were significant differences between the two generations only in the prepupal and pupal periods (*t*-test, after adjustment by the Bonferroni method: *p* > 0.05 for egg and larval periods; *p* < 0.05 for prepupal and pupal periods).

**Table 2:** The length of each development period of an egg, larva, and prepupa and pupa (day) in the summer and overwintering generations of *Dipogon sperconsus.*

Developmental stage	Sex	Summer generations			Overwintering generation		
		Mean ± SD	Range	(n)	Mean ± SD	Range	(n)
Egg	♀	2.17 ± 0.39	2–3	(12)	3.00 ± 0.53	2–4	(8)
	♂	2.50 ± 0.76	2–4	(8)	2.80 ± 0.45	2–3	(5)
Larvae	♀	4.96 ± 0.71	4–6	(23)	5.87 ± 1.28	4–9	(30)
	♂	4.23 ± 0.93	2–5	(13)	5.13 ± 1.20	3–7	(16)
Prepupa and Pupa	♀	16.0 ± 1.78	10–22	(64)	237 ± 17.1	204–276	(117)
	♂	14.9 ± 2.39	8–20	(41)	239 ± 17.4	200–276	(88)

The lengths of every developmental period of this species in the summer generation were almost the same as those of *D. sayi sayi* and *D. papago anomalus* in the summer generation (*D. sayi sayi*: egg period, 2 days; larval period, 5–6 days; prepupal and pupal periods, 14 days in Wisconsin, USA^8^; and egg period, 2 days; larval period, 5–7 days; prepupal and pupal periods, 13–16 days in males and 13–17 days in females in Plummers Island, Maryland, USA^2^; *D. papago anomalus*: egg period, 2 days; larval period, 5 days; prepupal and pupal periods, 18 days in Plummers Island^2^.

#### Life cycle and voltinism

**Figure 2:**
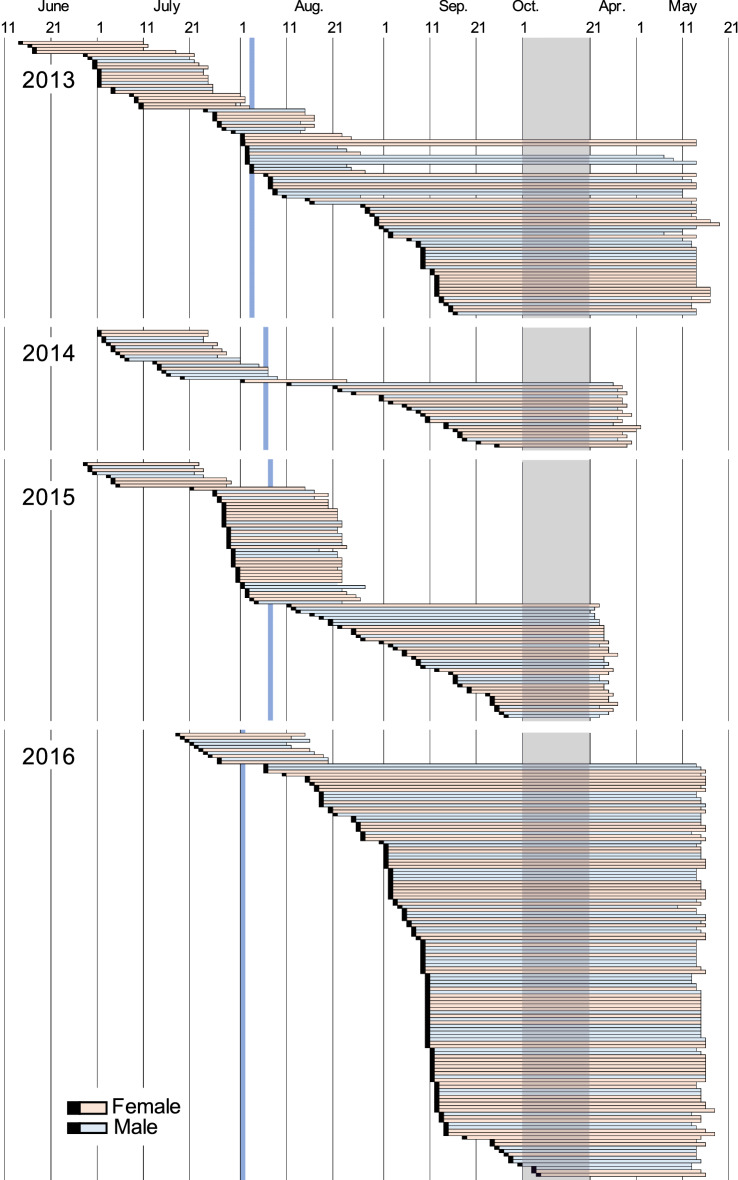
Developmental periods from egg to adult emergence based on the rearing data for individual wasps of *Dipogon sperconsus* during 2013–2017. Each horizontal bar shows the period from the estimated day of oviposition (indicated by the black beginning of bar) to the day of adult emergence (indicated by the end of bar) for an individual wasp (see Supplementary Tables S9–S12). Narrow blue areas show the 50% overwinter-egg days in each year (see text). A broad gray area represents the winter season.

**Figure 3:**
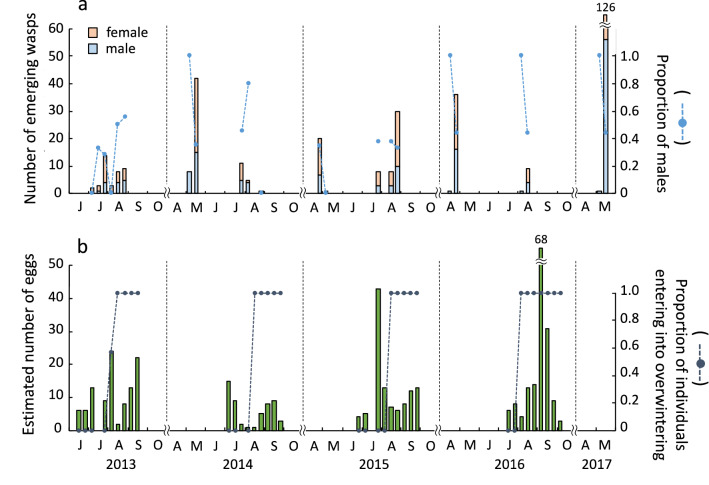
Seasonal changes in the numbers of newly emerging adults of *Dipogon sperconsus* and its eggs laid for 10 days of each month from 2013–2017. (**a**) The number of adult wasps emerging from rearing nests under room conditions (bars) and the male sex ratio of emerging wasps (blue circles) (see also Supplementary Table S13). (**b**) The estimated number of eggs laid by female wasps in nests (bars) and the proportion of the eggs that would develop into overwintering individuals among all eggs for each period (gray circles) (see also Supplementary Table S14).

To examine the seasonal changes in nesting activities, we illustrated the developmental periods of individual wasps (Fig. 2) and the number of emerging wasps and the egg production for every 10 days of each month (Fig. 3). The egg production was calculated from the oviposition days estimated from the developmental stages and rearing data for individuals in nests collected over four years (Supplementary Tables S9–S12). Oviposition of *D. sperconsus* and unknown *D*. spp. began from mid-June to early July and continued until mid-September to early October (Supplementary Figure S3). Because the developmental period from egg to adult emergence is about 21–23 days for the summer generations (Table 2, Supplementary Tables S9–S12), it is theoretically possible that at most four or five generations emerge into adults over the whole nesting season (96 days in 2013, 88 days in 2014, 110 days in 2015 and 103 days in 2016; Supplementary Tables S9–S12). However, the actual number of generations per year appeared to be smaller: three in 2013 and 2015, and two or three in 2014 and 2016 (Figs. 2, 3a, Supplementary Figure S3). This discrepancy between the expected and observed number of generations could be explained by the fact that most of the eggs laid after early August became the overwintering generation (Fig. 2). We here call those eggs, the ‘overwintering’ eggs. The ‘overwintering’ eggs started appearing at the beginning of August (possibly late July, but no records) and sharply increased to become 100% early August (Fig. 3b).

The 50% ‘overwintering’ egg days were August 3 in 2013, August 6 in 2014, August 7 in 2015, and August 1 in 2016. These switching day varied about a week, but all fell in early August. Because there were only about 50 days available for nesting females from mid-June (earliest nesting activity) to early August (switching day), up to two generations could emerge before producing the overwintering generation. Thus, the present population has a life history with two or three generations per year, depending on how many generations emerge into adults before early August. Comparing the switching day (August 3 in 2013, August 6 in 2014, August 7 in 2015, August 1 in 2016) with the egg production peak in four years, the switching day was found to be around the second peak of oviposition in 2013, between the first and second peaks in 2014, and after the second peak (before the third peak) in 2015 (Fig. 3b). The switching day appeared to be immediately after the first peak in 2016 (Fig. 3b). However, if we regard a small rise in the cumulative number of eggs in the late June of 2016 as the first peak (Supplementary Figure S3), the switching day is immediately after the second peak in the year. These results indicate that in 2013, 2015 and 2016, the second generation mostly produced eggs for the third generation and a few eggs for the overwintering generation (i.e., most individuals are trivoltine), while in 2014 the second generation produced many eggs for the overwintering generation (i.e., most individuals are bivoltine).

The life cycle of this species typically seemed to proceed as follows: Wasps of both sexes in the overwintering (first) generation emerged almost simultaneously for approximately 10 days from late April to mid-May. The first-generation wasps laid eggs from mid-June to mid-July. There is about a two-month blank between emergence and oviposition in these ‘overwintering’ wasps. Hatched individuals of the eggs emerged from early July to early August (second generation). The offspring of the second generation had two possibilities. (1) Emerging in that summer becoming the third generation (when the second-generation females laid eggs before early August, these eggs became adults around mid- and late August), all the offspring of which entered into the overwintering stage (i.e., trivoltine). (2) Overwintering and emerging in the following year (when the second-generation females laid eggs in or after early August, these eggs became hibernating prepupae) (i.e., bivoltine). Thus, the voltinism of this species in the study site is variable between bivoltine and trivoltine, depending on the proportion of the eggs laid before early August by the second-generation females. We, however, could not exclude the possibility that a few early-developing individuals of the third generation emerged into adults before early August and that the fourth-generation adults appeared within a year (i.e., multivoltine). In summary, this species is plurivoltine. Similar plurivoltine was reported in *D. sayi sayi*. This wasp is bivoltine in Ontario^9^ and New York, USA^2^, and probably trivoltine in Washington, D.C., USA^2^.

There was a tendency for males in the overwintering generation to emerge earlier than females, but such a tendency was not noticeably demonstrated in the subsequent generations (Fig. 3a, Supplementary Table S13). The overall sex ratio was 0.43, slightly biased toward females. In *D. sayi sayi*, the sex ratios were 0.73 (1961), 0.5 (1962) and 0.38 (1963) in the summer generation and 0.38 (1961) and 0.5 (1962) in the overwintering generation in Northwestern Ontario^9^; 0.78 (35:10) in Washington County, USA^16^; and 0.29 (31:76) in Plummers Island^2^.

To elucidate the factors affecting the switching day, we investigated the temperature that wasps experienced in the preceding period. For the temperature, the meteorological data from Sanda, Hyogo Pref. (the nearest weather-monitoring station to the present investigation site) were used (Japan Meteorological Agency: http://www.data.jma.go.jp).

**Figure 4:**
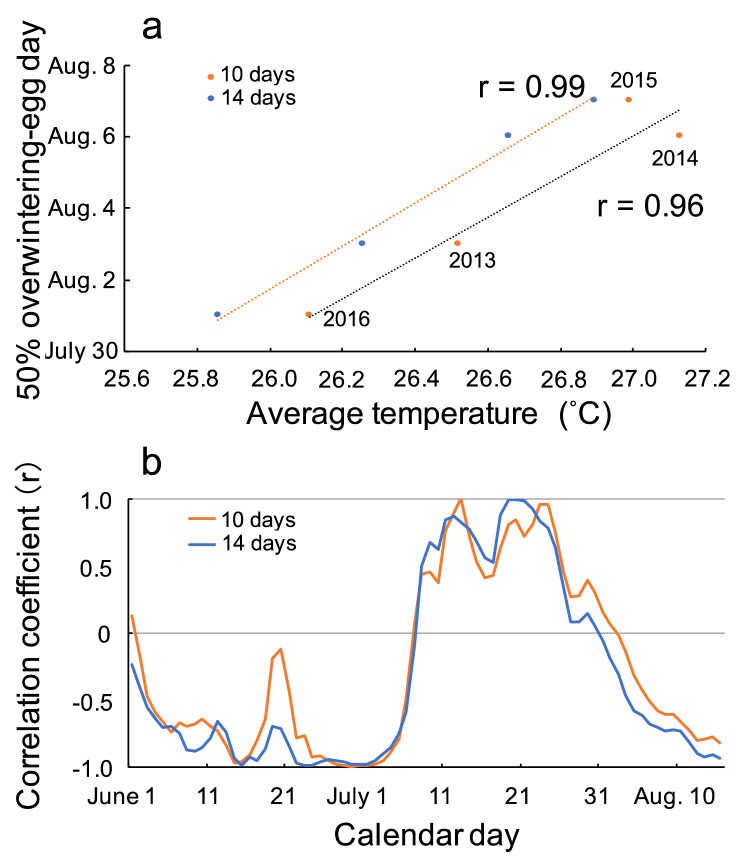
Relationship between temperature and the estimated day at which 50% of eggs laid on a given day would become overwintering individuals (the “switching” day). (**a**) Correlation between the mean temperature for 10 days from July 23 to August 1 or 14 days from July 19 to August 1 and the switching day. (**b**) Daily change in correlation coefficients between every 10 (14)-day mean temperature and the switching day each year. The curved line indicates that there was a high correlation between the mean temperature in mid- and late July and the switching day in 2013–2016.

Figure 4a shows the relationship between the average temperature from July 23 to August 1 (the 10-day) or from July 19 to August 1 (14-day) period before the switching day and the switching day in each year (see also Supplementary Table S15). Note that the 10-day average temperature is calculated from the 10-day cumulative temperature divided by the 10-day (days) and 14-day average temperatures in the same manner. Figure 4a indicates that there was a very high correlation between them (10 days: *r* = 0.96, *t* = 4.92, *p* = 0.03; and 14 days: *r* = 0.995, *t* = 14.9, *p* = 0.004). The percentage of overwintering eggs was thus negatively correlated with the preceding cumulative temperatures that the female experienced. This correlation indicated that this wasp shows increased voltinism from bivoltine to trivoltine when the cumulative temperature is high (i.e., in a hotter summer). This high correlation suggested that overwintering is determined by a female herself when she lays an egg. This phenomenon, wherein the temperature experienced by a female influences the induction of diapause in her offspring, is known in some hymenopterous insects, e.g., *Trichogramma*^27^. There was a high correlation period in mid- to late July (Fig. 4b, Supplementary Table S16), indicating that the timing of switching physiological conditions of female wasps is dependent on the average temperature in this critical period. To confirm whether temperature conditions in the field practically influence the life cycle of *D. sperconsus*, further studies will be needed.

The emergence days of the overwintering generations were clearly different among years: most wasps emerged before May 1 in 2014 and 2015, while most wasps emerged after May 10 in 2013 and 2016 (Fig. 2). April mean temperature in Sanda, Hyogo Pref. was higher in 2014 (13.7 °C) and 2015 (14.1 °C) than in 2013 (11.8 °C) and 2016 (12.8 °C) (Japan Meteorological Agency: http://www.data.jma.go.jp). Thus, such higher air temperatures during the post-diapause period in 2014 and 2015 may have triggered the earlier emergence of the overwintering generations.

There was up to a two-month-duration between the spring emergence period (overwintering wasps in the laboratory: Fig. 3a) and the initial nesting period (wasps in the field: Fig. 3b). One of the reasons for such a long pre-nesting period of the overwintering wasps is that the overwintering wasps indoors emerged much earlier than those in the field, because the temperature condition indoors was warmer than that in the field. If it is the fact, this period may be actually shorter in the field than indoors. Another reason for the long-delayed oviposition may be due to the loss of energy during the winter; these wasps may have to collect energy and nutrients for egg maturation greatly. In fact, in some spider wasps, we often observed females hunt spiders solely for their own consumption (sting the spiders, masticate them with their mandibles, and ultimately abandon them in situ)^28^. We need further studies to determine when overwintering wasps emerge in the field and what physiological conditions are necessary for egg maturation of overwintering wasps.

**Figure 5:**
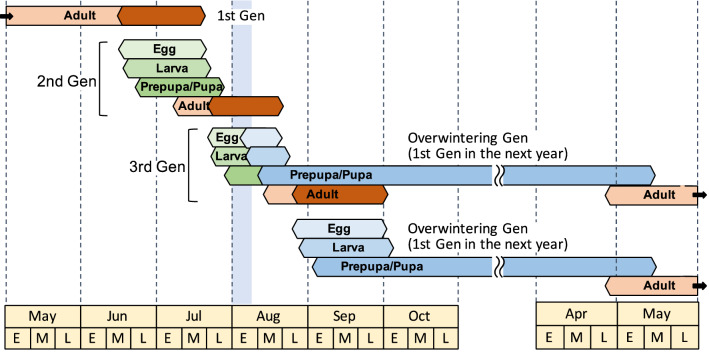
A schematic diagram of the life cycle of *Dipogon sperconsus* in Takarazuka, Hyogo, Japan. The orange parts of the adult spans represent nesting periods. 1st, 2nd, 3rd and Overwintering Gens represent the first, second, third and overwintering generations in a year, respectively. There is a short critical period during early August (blue area) in which wasps of the third generation would develop into adults either in the summer (summer generation) or in the next spring via diapause of prepupae (overwintering generation). Part of the third generation and almost all of the overwintering generation (deep blue part) become hibernating prepupae, i.e., the wasps exhibited a partial trivoltine life cycle. Because the beginning of nesting activity in the first generation varied according to year, the proportion of adults emerging in the summer to those emerging in the following year changed annually, i.e., wasps were almost all trivoltine in some years and almost entirely bivoltine in other years. A possibility of quadvoltine is not shown in this figure (see text).

We integrated all these findings into a schematic illustration showing the life cycle of *D. sperconsus* in the present population (Fig. 5). This is the first record of the life history schedule of a spider wasp based on the consecutive daily surveys (Figs. 2, 3, 4).

### Nesting activity

#### Cell productivity per day

The results of the consecutive daily investigations from 2014–2016 showed that 68 canes (2.7%; 68/2565*100) were utilized by *Dipogon sperconsus* among all 2565 canes installed in 2014 to 2016 (Supplementary Table S1 and S17). We also counted how many cells were constructed in a day: Out of a total of 68 nests, a single cell/day was found in 39 examples; two cells/day in 27 examples; three cells/day in seven examples; and four cells/day in two examples (Supplementary Table S17). Thus, the wasps constructed on average 1.63 cells/day (1–4, SD =  ± 0.77 cells/day). We should note that some nests were already found with one or more cells at the time of the weekly routine surveys. We excluded these records from the above data because we could not determine the starting dates for the analysis of cell production (Supplementary Table S17). This result was comparable to the three cells constructed in a single day by *D. sayi sayi*^2^.

#### Prey spider species and sizes

One hundred and two spiders were identified as *D. sperconsus* prey over the four years. These comprised 74 coelotids, 21 agelenids, 5 segestriids and 2 thomisids (Table 3). The total of the former two occupied 93% of all samples.

**Table 3:** Comparison of the cephalothorax width (mm) of the prey spiders on which *Dipogon sperconsus* laid eggs.

Prey spider	Sex	Wasp laid						*t* test
		Female-destined egg			Male-destined egg			
		Mean ± SD	Range	(n)	Mean ± SD	Range	(n)	
Coelotidae	Female	2.45 ± 0.34	1.8–3.1	(20)	1.95 ± 0.45	1.5–3.1	(20)	***
	Male	2.74 ± 0.32	2.0–3.1	(26)	2.15 ± 0.30	1.8–2.6	(8)	***
Agelenidae	Female	2.18 ± 0.29	1.6–2.5	(10)	2.08 ± 0.07	2.0–2.1	(2)	NS
	Male	2.70 ± 0.40	2.4–3.1	(2)	2.60 ± 0.43	1.9–3.0	(7)	NS
Segestriidae	Female	2.30 ± 0.19	2.0–2.5	(4)	1.94	1.9	(1)	–
Thomisidae	Female	–	–	(0)	2.80 ± 0.55	2.4–3.2	(2)	–

***Indicates the significance level at p < 0.001.

For the coelotid spiders, 40 females and 34 males were used as prey, which shows that there was no significant sex bias in prey (z = 0.70, *p* > 0.1). Among the 40 coelotid female spiders, the sex ratio of wasp eggs was even: 20 female wasp eggs and 20 males. However, the female spiders on which female wasp eggs were laid were significantly greater in cephalothorax width than those on which male eggs were laid (t = 3.98, *p* < 0.001; Table 3). When male spiders were considered, significant more female eggs were laid than male eggs (z = 3.09, *p* < 0.01). The male spiders on which female eggs were laid were also significantly larger in cephalothorax width than those on which male eggs were laid (t = 4.61, *p* < 0.001). In other families of spiders, there was also a tendency for the spiders bearing female eggs to be larger than those bearing male eggs, but the differences were not significant (Table 3). These results indicated that a female wasp lays female or male eggs selectively depending on prey spider size. This habit is also recognized by an ichneumonid ectoparasitoid, *Zatypota albicoxa* (Walker), parasitic on *Parasteatoda tepidariorum* (L. Koch)^29^.

#### Unutilized prey spiders

Occasionally trap nests included cells with a prey spider but without a wasp egg (“N” in Supplementary Table S17). These examples totaled 103 cases (6 in 2013, 12 in 2014, 32 in 2015 and 53 in 2016), which occupied 16.5% (103/623*100) of all cells. Apparently, these were not due to wasp death immediately after prey carriage because all of the cells containing a spider without an egg were partitioned or closed. The frequency of such cells tended to increase in the periods when many cells were constructed (coefficient of correlation between these factors per 10 days: *r* = 0.69, *p* < 0.001), but the reason for this was unknown.

### Comparison with other species

As stated in the introduction, most of the tube-nesting species in Pompilidae belong to *Auplopus* and *Dipogon*^2,3^. The nesting behavior of both genera, however, are fundamentally different. Species of *Auplopus* build barrel-shaped mud cells in series in tubes (building type)^1,30^, while species of *Dipogon* construct a series of cells that are partitioned and closed by stuffing mud particles, sawdust and other materials (renting type)^1,10^. The effects of the difference on wasps’ survival are still not clear.

Fragmentary data on the number of cells in a nest, the length of each developmental period and the sex ratios have been reported in some pompilid species^2,8,9,16^. However, precise information on nesting ecology is still lacking, e.g., seasonal changes in the numbers of newly emerging adults and voltinism. The current report presents the first detailed data on the nesting ecology that can be used for the comparisons with other species in future studies.

## Materials and methods

### Investigation site

Our study site was a narrow area approximately 1,500 m in length and 100 m in width along a forested path, situated in Kirihata, Takarazuka City, Hyogo Pref., Japan (34° 51′ 31″ N, 135° 20′ 13″ E), at approximately 350 m above sea level. This area was fundamentally old pine tree forest (Fig. 1a). The canopy layer of the forest was dominated by Japanese red pine trees *Pinus densiflora*, most of which stood dead and a few of which were living and sporadically distributed; the understory layer was dominated by *Quercus serrata*, *Cerasus jamasakura* and *Ilex pedunculosa*, the shrub layer by *Eurya japonica* and *Lindera umbellata*, and the herb layer by *Tripterospermum japonicum* and *Blechnum niponicum*.

### Methods

Trap nests were dried bamboo canes 200 mm long with one end closed by the node (Fig. 1b). The inside diameter of the canes greatly influences the species composition of trap nest users^23^. We used canes with inside diameters of 10–13 mm (M-size), 7–10 mm (S-size) and 4–7 mm (XS-size) but not 13–16 mm (L-size) because the utilization rate of the L-sized cane is low for species of *Dipogon*^24–26^ The above three types of canes were bounded vertically in descending order and repeated five times, with garden wires covered with vinyl; hence, a bundle of 15 canes was arranged in a single layer as a screen (Fig. 1b). We attached bundles of canes (trap nests) to randomly selected tree trunks, approximately 1.5 m above the ground, separating each by approximately 10 m. A group of traps in each year were installed 50–100 m apart from one another.

The investigations were conducted from 2013 to 2016: 32 traps were set up in 2013 and 2014 each, 70 traps in 2015, and 69 traps in 2016 (Supplementary Table S1). These trap nests were placed in the forest from early June to late October every year except in 2014. In 2014, the traps were set up in early May, and the survey was initiated soon after that. The yearly nesting periods calculated from the first day to the last day when nesting activity was observed ranged from 87 (2014) to 119 days (2015) (Supplementary Table S1). The first observed nesting day was July 1 (2014), and the last nesting day was October 19 (2015).

In 2013, on the first day of the yearly survey, we installed trap nests at the site. After the installation of trap nests, weekly unless the weather (heavy rains) prevented, we collected all bamboo traps and replaced them with similar new traps, usually early in the morning. Next, we brought the traps back to the laboratory and split them open to check for the presence or absence of *Dipogon* cells (Fig. 1c–e). When the cells were detected, their arrangement and the developmental stages of individuals were recorded. The wasp eggs (Fig. 1d) or larvae (Fig. 1e) were reared to adults in the following manner (when the adults emerged, we were able to confirm their species and sexes for the first time): each spider with an egg or larva was moved to a small plastic case (L 1.5 cm × W 2 cm × H 1 cm), which was then kept in a north-facing room, and the individuals were reared up to the prepupal stage at room temperature. All prepupae were moved to small glass vials loosely plugged with caps and were kept in the room. Adults emerging within the year were classified as the second and third generations. The remainder of prepupae in the vials were kept in a barn without a heater in winter and were then moved back to the laboratory after March. Adults emerging after winter were classified as the first generation of the next year. The emerging adults were mounted for identification. When a dead individual was detected in the nest from which identified adults also emerged, the former was identified as the same species as the latter. This is based on the fact that there were no examples in which wasps of two species emerged from the same cane in the study site, as stated at the top of the results and discussion. In rearing, the condition of each individual, e.g., hatching, spinning, emerging or dead, was checked at a fixed time every day. When a spider without an egg or larva or a spider with a dead individual were detected in a cell, these spiders were immersed in 70% ethanol for identification.

From 2014 to 2016, after each weekly survey (as in 2013), consecutive daily surveys of the trap nests were conducted. In these surveys, we inserted straw (a stalk, culm or stem of a grass/bamboo) into each cane to probe the presence of cells. If there was a partition (cells), the straw should not have reached the innermost recesses of the cane. We were able to notice the presence or absence of the partition by the length of the insertable part of the straw. The consecutive daily surveys were carried out in a following manner. In the weekly survey, if we found *Dipogon* cells in traps, we recorded the trap numbers and localities (wasp eggs on spiders were reared as explained above). At the dawn of the following day, by the straw probing, we checked several canes above and below the cane where *Dipogon* cells were detected the day before. If we found a cane containing a newly constructed partition, we pulled it out from the bundle and replaced it with a similar new one. The wasp-containing canes were brought to the laboratory, and eggs on spiders in the cells were reared. We followed the same procedure until no nesting was confirmed in these traps for three days. About seven days later from the last weekly-survey day (the practical day interval between the weekly surveys varied from six to eight days, depending on weather), we resumed the unit of surveys from the start (complete exchange of all trap bundles at the beginning, and individual exchange of wasp-containing canes on following several days) until no nesting was confirmed in traps for a full week (the total days when we checked trap nests in both weekly surveys and consecutive daily surveys were 20 days in 2013, 50 days in 2014, 43 days in 2015 and 28 days in 2016) (Supplementary Table S1).

From the above surveys, the following data were obtained: (1) egg production per day; (2) the egg period from the estimated oviposition day to the hatching day (when we first detected an egg in a cell on a certain day in the daily survey, we identified its oviposition day as the previous day); (3) the larval period from the hatching day to the initial day of spinning; and (4) the prepupal and pupal periods from the spinning day to the emerging day. The egg production per day was directly found by the follow-up daily surveys, except for the first day in the weekly surveys. The average period of the egg stage was counted as the average length from the day before egg-detecting day to the hatching day. The average larval period was calculated from the larval periods of all collected eggs. From these average egg and larval periods for each sex, emergence period and generation, we estimated the oviposition day for individuals collected at the larval stage. For example, when we found a larva in a collected nest and recorded the spinning day of the larva, we estimated its oviposition day by counting back to include the total time of the egg and larval periods from that day (see Table 2). Based on the estimated oviposition days, we calculated the numbers of eggs laid for 10 days of each month and the period from oviposition day to emergence day for each individual, and then investigated the seasonal changes in the number of eggs laid by female wasps and the voltinism of this species.

## Supplementary Information

Supplementary Information.
