# Supplementary material for: Life history and nesting ecology of a Japanese tube-nesting spider wasp Dipogon sperconsus (Hymenoptera: Pompilidae)
Source: Sci Rep. 2021 Jun 17;11:12810. doi: 10.1038/s41598-021-92124-z (PMC8211671; doi:10.1038/s41598-021-92124-z)
Supplement: Supplementary file 1 — Supplementary Information. [file 41598_2021_92124_MOESM1_ESM.pdf]

Supplementary Information for
Life history and nesting ecology of a Japanese tube-nesting spider wasp (Hymenoptera: Pompilidae)

Yutaka Nishimoto[1], Akira Shimizu[2,3,4], Jin Yoshimura[3,4,5,6], Tomoji Endo[7]

[1]Takarazuka, Hyogo, 669-1211 Japan,
[2]Department of Biological Science, Tokyo Metropolitan University, Hachioji, Tokyo, 192-0397 Japan,
[3]Research Institute of Evolutionary Biology, Inc., Setagaya-ku, Tokyo, 158-0098 Japan,
[4]University Museum, the University of Tokyo, Bunkyo-ku, Tokyo, 113-0033 Japan,
[5]Department of International Health and Medical Anthropology, Institute of Tropical Medicine, Nagasaki University, Nagasaki, 852-8523 Japan,
[6]Marine Biosystems Research Center, Chiba University, Uchiura, Kamogawa, Chiba, 299-5502 Japan and
[7]Department of Biosphere Sciences, School of Human Sciences, Kobe College, Nishinomiya, Hyogo, 662-8505 Japan

Contents:
Supplementary Figure S1-S3
Supplementary Tables S1-S17

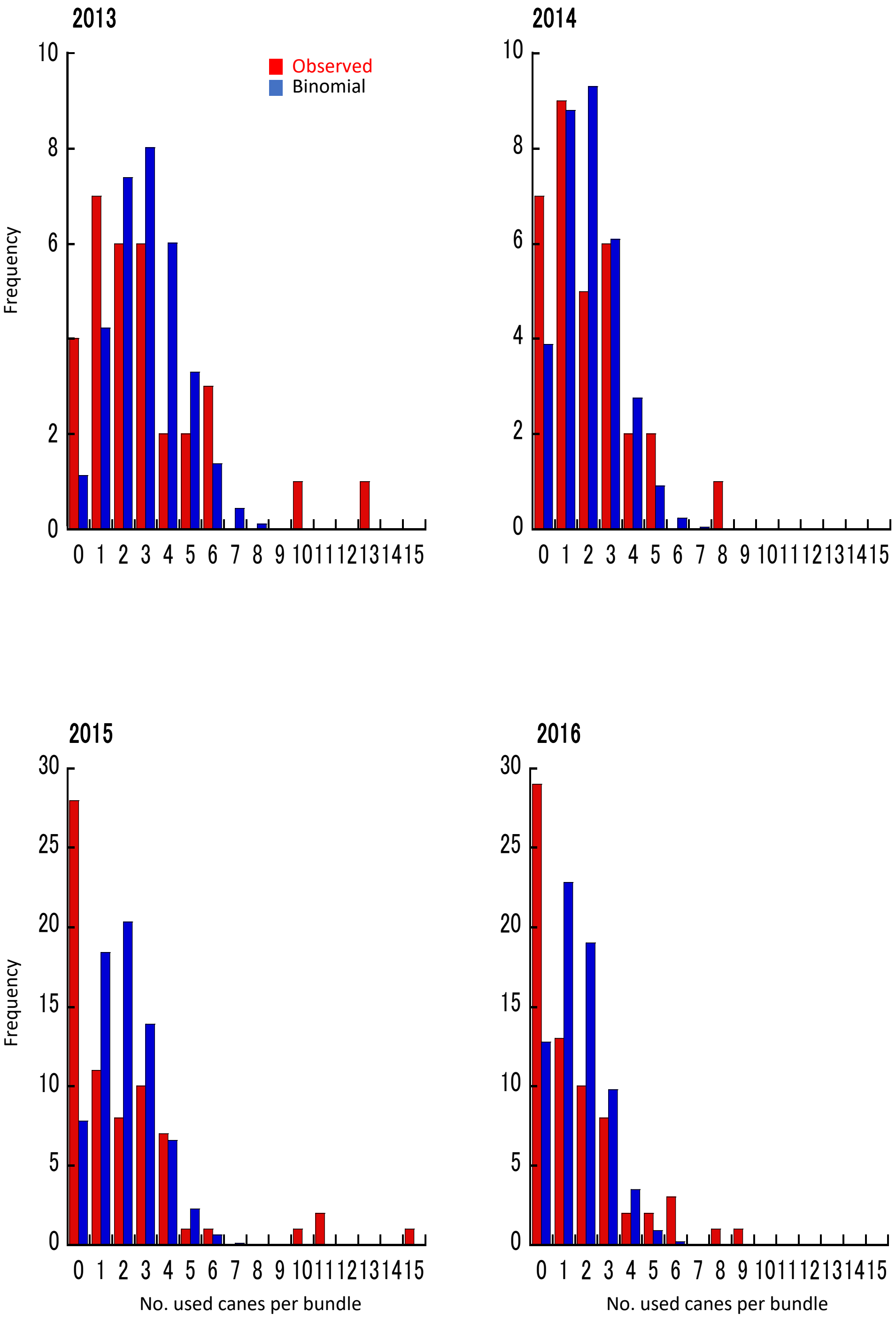

Supplementary Figure S1. Yearly frequency distributions of the used canes by *D. sperconsus* and/or *D.* spp. per cane bundle during 2013–2016. The binomial distributions are the theoretical expectation assuming random uses of canes with the same yearly mean. Results indicate aggregated distributions for 2015 and 2016, a strong tendency for aggregated distribution for 2013 and a tendency for aggregated distribution for 2014.

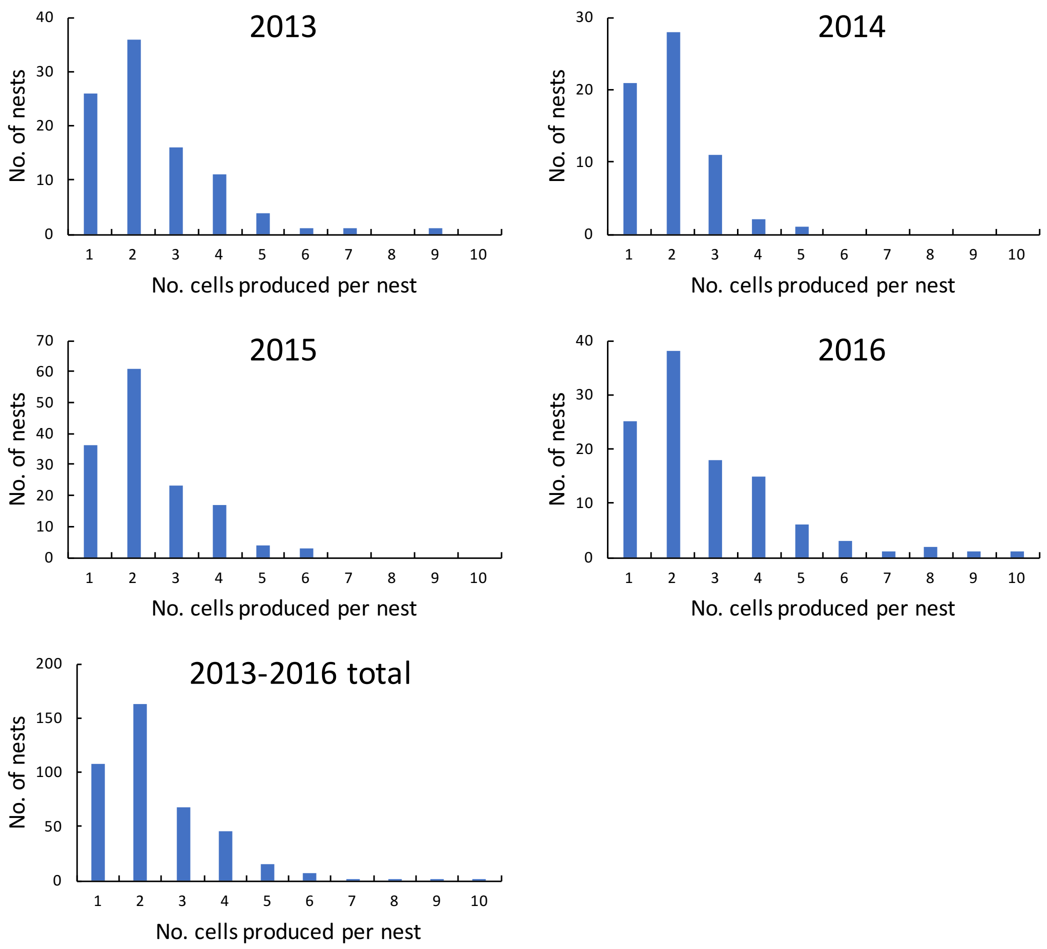

Supplementary Figure S2. Yearly frequency distributions of the number of cells constructed by *Dipogon sperconsus* and/or *D.* spp. per nest from 2013–2016.

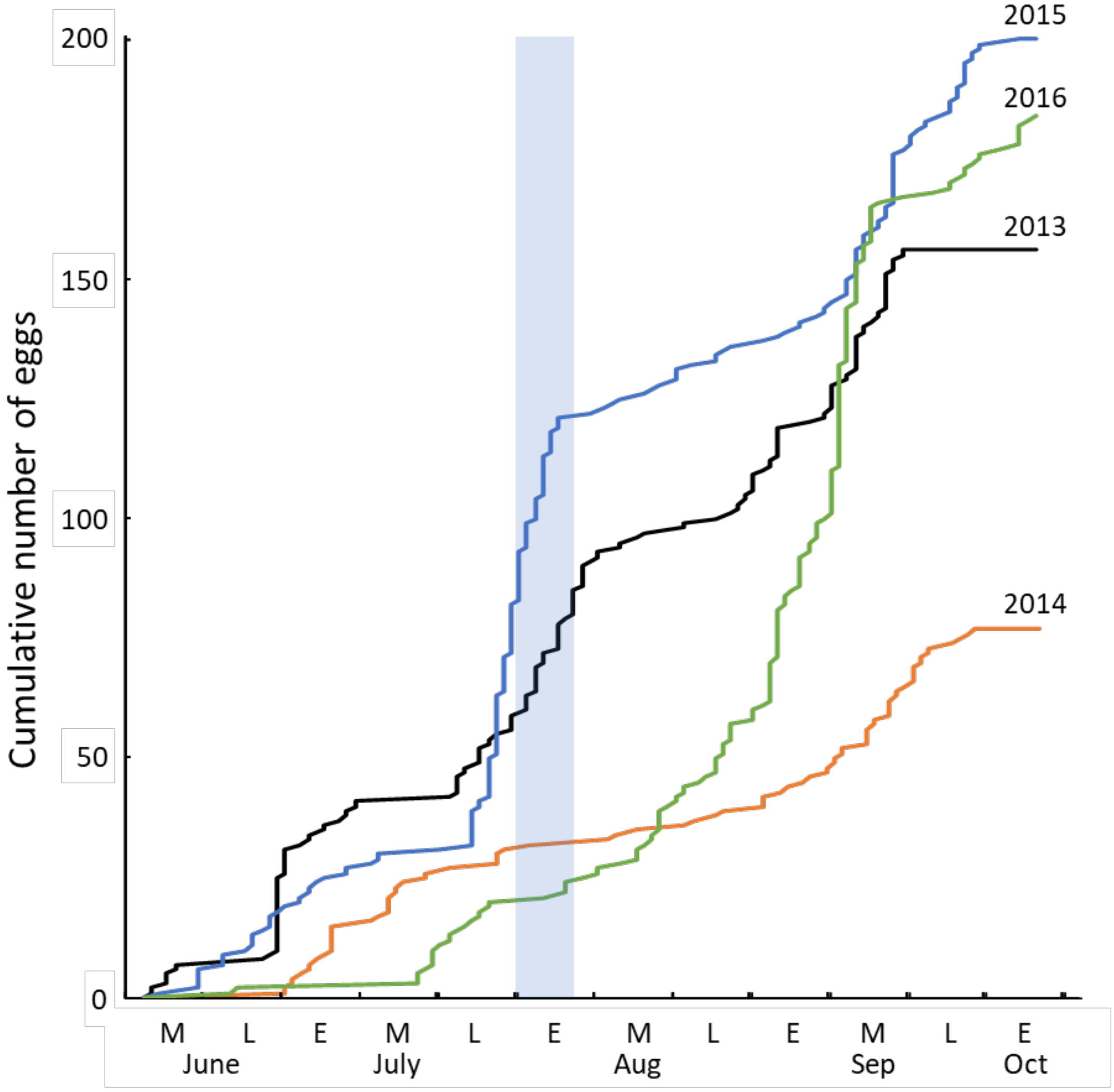

Supplementary Figure S3. Cumulative number of eggs laid by *Dipogon sperconsus* and unknown *D*. spp. along the nesting season during 2013-2016. Daily numbers of eggs were estimated from the developmental stages of individuals in nests collected through the weekly and consecutive field surveys (see the method). The cumulative numbers of eggs include data on the individuals that died during rearing in order to evaluate the temporal change in egg production as accurately as possible (Supplementary Tables S9–S12). The range of the switching days in the four years is shown as a blue area in early August (see the text).

Supplementary Table S1. The trap nest investigations conducted in Takarazuka, Hyogo (2013–2016)

Year	2013[*1]	2014	2015	2016	Total
No. of cane bundles	32	32	70	69	203
No. of canes	480	480	1050	1035	3045
Investigation period (days)	June 11–Oct. 21 (132)	May 8–Oct. 28 (173)	June 5–Oct. 28 (145)	June 4–Oct. 21 (139)	589
Investigation days in field[*2]	20	50	43	28	141
Yearly nesting period (days)[*3]	June 17–Sept. 25 (100)	July 1–Sept. 26 (87)	June 22–Oct. 19 (119)	June 18–Oct. 14 (118)	424

[*1]We did not conduct the consecutive daily investigations in 2013.

[*2]The total days include the days when we checked trap nests in both weekly surveys and consecutivel daily surveys.

[*3]The yearly (seasonal) nesting period is calculated from the first day to the last day when nesting activity was observed in each year.

Supplementary Table S2. The numbers of the nests of wasps other than *Dipogon* , bees, parasitic wasps and flies collected in the present trap nest investigation.

	2013	2014	2015	2016	Total
Other Pompilidae					
Auplopus carbonarius (Scopoli)	24	21	62	92	199
Vespidae					
Discoelius zonalis (Panzer)	74	8	11	117	210
Anterhynchium flavomarginatum (Smith)	40		7	1	48
Sphecidae					
Isodontia harmandi (Pérez)	74	5	1	23	103
Crabronidae					
Crabronine spp.	1				1
Trypoxylon spp.	18		12	6	38
Megachilidae					
Megachile sculpturalis Smith	12	5		2	19
Megachile spp.	3	1		2	6
Euaspis basalis (Ritsema)*	2				2
Colletidae					
Hylaeus spp.	2	2			4
Eulophidae					
Melittobia spp.**	3	1			4
Parasitic Diptera***	2				2

*bood-parasitized *Megacile sculpturalis* .

**parasitized *Auplopus carbonarius* (3 cases) and *Isodontia harmandi* (1 case).

***parasitized *Trypoxylon* sp. (1 case) and *Anterhynchium falavomarginatum* (1 case).

Supplementary Table S3. Distribution of the nests and cells of *Dipogon sperconsus* and *D*. spp. in 2013.

Site	Cane bandle No.	No. of *D. sperconsus* nests	No. of unknown *D*. spp. nests	No. of nests of *D. sperconsus* and/or *D*. spp.	Frequency distribution of nests containing 1–9 cells of *D. sperconsus* and/or *D*. spp.									Total No. of cells
					1 cell	2 cells	3 cells	4 cells	5 cells	6 cells	7 cells	8 cells	9 cells	
A	1	5	1	6	1	1	2	2						17
	2		2	2	1		1							4
	3													
	4	1	1	2		1	1							5
	5	1	1	2		1				1				8
	6	1		1	1									1
	7	3	3	6	2	1		2	1					17
	8													
	9	2	1	3	1	2								5
	1	2	2	4	2	1	1							7
	11	1	2	3		3								6
	12		2	2	1		1							4
	13	3	3	6		1	2	1	1				1	26
	14	2	3	5	1	2		1			1			16
	15													
	16		1	1		1								2
B	1	3	1	4	2	2								6
	2	6	7	13	3	3	4	2	1					34
	3	2	3	5		3	1		1					14
	4	7	3	10	3	6	1							18
	5	1	1	2		2								4
	6		1	1	1									1
	7		1	1	1									1
	8		1	1				1						4
	9	1	2	3	2	1								4
	1		2	2	1		1							4
	11	1		1		1								2
	12		3	3	1	1	1							6
	13	1	2	3		2		1						8
	14													
	15		1	1	1									1
	16	1	2	3	1	1		1						7
Total		44	52	96	26	36	16	11	4	1	1	0	1	232

Supplementary Table S4. Distribution of the nests and cells of *Dipogon sperconsus* and *D*. spp. in 2014.

Site	Cane bandle No.	No. of *D. sperconsus* nests	No. of unknown *D.* spp. nests	No. of nests of *D. sperconsus* and/or *D.* spp.	Frequency distribution of nests containing 1–5 cells of *D. sperconsus* and/or *D*. spp.					Total No. of cells
					1 cell	2 cells	3 cells	4 cells	5 cells	
C	1		1	1		1				2
	2									0
	3	1		1			1			3
	4		1	1	1					1
	5	2	1	3	1	1	1			6
	6	1	1	2	1	1				3
	7	1		1	1					1
	8		3	3	2	1				4
	9	2	1	3	1	1		1		7
	10	4		4	1	3				7
	11	1	1	2		2				4
	12	3		3	2		1			5
	13		1	1		1				2
	14	1	4	5	3	2				7
	15		1	1			1			3
	16		1	1			1			3
D	1	2	1	3	1	2				5
	2	2		2	1	1				3
	3		1	1	1					1
	4	1		1	1					1
	5	1	1	2	1	1				3
	6	3	1	4	1	3				7
	7	7	1	8	1	3	2	1	1	22
	8									0
	9	1	1	2	1		1			4
	10									0
	11									0
	12	3	2	5		3	2			12
	13	2	1	3		2	1			7
	**14**									**0**
	**15**									**0**
	**16**									**0**
Total		38	25	63	21	28	11	2	1	123

Supplementary Table S5. Distribution of the nests and cells of *Dipogon sperconsus* and *D*. spp. in 2015.

Site	Cane bandle No.	No. of *D. sperconsus* nests	No. of unknown *D.* spp. nests	No. of nests of *D. sperconsus* and/or *D.* spp.	Frequency distribution of nests containing 1–6 cells of *D. sperconsus* and/or *D.* spp.						Total No. of cells
					1 cell	2 cells	3 cells	4 cells	5 cells	6 cells	
E	1										0
	2										0
	3										0
	4		1	1				1			4
	5	1		1			1				3
	6	6	9	15	2	10	1	3			37
	7	1		1						1	6
	8	4	7	11	3	6	1	1			22
	9	3	1	4	1	2		1			9
	10	2	1	3		1	1	1			9
	11	2	1	3	1	1			1		8
	12	1	2	3		1	2				8
	13	1	1	2	1	1					3
	14	1	1	2		1		1			6
	15	1	3	4	1	1	1	1			10
	16	1	2	3	1	1	1				6
F	1										0
	2										0
	3	1	3	4		3			1		11
	4	1		1	1						1
	5										0
	6	2	1	3	1			2			9
	7										0
	8	1	1	2	1	1					3
	9										0
	10	1	3	4	1	1		1		1	13
	11	5	6	11	4	3	3	1			23
	12	1	3	4	1	2	1				8
	13		1	1			1				3
	14	1	1	2			1	1			7
	15	2	1	3	1	1		1			7
G	1										0
	2										0
	3										0
	4										0
	5		3	3		3					6
	6	1		1		1					2
	7										0
	8										0
	9										0
	10	1	3	4	1	1	2				9
	11		3	3		3					6
	12	2		2		1	1				5
	13	2	4	6		4	2				14
	14	1		1					1		5
	15										0
	16	2		2		1		1			6
H	1										0
	2	5	5	10	5	4			1		18
	3										0
	4	1	1	2		2					4
	5										0
	6	3		3	1		1			1	10
	7	1		1				1			4
	8		5	5	4	1					6
	9										0
	10	2	1	3	2		1				5
	11										0
	12										0
	13	1	1	2	1	1					3
	14	1	3	4	1	2	1				8
	15										0
	16										0
	17		1	1		1					2
	18										0
	19										0
	20	1		1			1				3
I	1										0
	2										0
	3		1	1	1						1
Total		63	80	143	36	61	23	17	4	3	333

Supplementary Table S6. Distribution of the nests and cells of *Dipogon sperconsus* and *D* . spp. in 2016.

Site	Cane bandle No.	No. of *D. sperconsus* nests	No. of unknown *D.* spp. nests	No. of nests of *D. sperconsus* and/or *D.* spp.	Frequency distribution of nests containing 1–10 cells of *D. sperconsus* and/or *D* . spp.										Total No. of cells
					1 cell	2 cells	3 cells	4 cells	5 cells	6 cells	7 cells	8 cells	9 cells	10 cells	
J	1	6		6	3		2	1							13
	2	4	5	9	2	3	2	1	1						23
	3	1	1	2		1		1							6
	4	3	3	6	2	2	1					1			17
	5	6	2	8	1	3		1	2	1					27
	6														0
	7														0
	8														0
	9														0
	10	1		1			1								3
	11														0
	12	2	1	3		2				1					10
	13														0
	14														0
	15	1	1	2	1			1							5
	16		1	1		1									2
K	1	2		2		1	1								5
	2														0
	3														0
	4	1		1		1									2
	5	1	1	2		1		1							6
	6	1		1				1							4
	7														0
	8														0
	9														0
	10														0
	11	2	1	3			2	1							10
	12	1		1				1							4
	13														0
	14														0
	15	1		1				1							4
	16														0
L	1						1								0
	2	1		1											3
	3					1					1				0
	4	1	1	2		2									9
	5	1	1	2		3	1								4
	6	2	2	4											9
	7					1	1			1					0
	8	3		3	2										11
	9	2		2											2
	10														0
	11						1	1	1						0
	12	3		3	2	1									12
	13					1									0
	14				2	2			1				1		0
	15					2	1								0
	16	3		3	1	1	1								4
	17	1		1	1	1								1	2
	18	4	2	6											20
	19	2	1	3	2	1		1				1			7
	20	2	1	3	1		1	1	1						6
	21	2	1	3	1	1									13
M	1				1	3		1							0
	2	2	3	5			1								16
	3	4		4											13
	4	1	1	2		1									3
	5	4	1	5		1									11
	6	1		1		1									3
	7				1			1							0
	8		1	1											2
	9	1		1											2
	10		1	1		1									2
	11	1	1	2	1			1							5
	12														0
	13														0
	14	2		2	1		1								4
	15		1	1	1										1
	16														0
Total		76	34	110	25	38	18	15	6	3	1	2	1	1	305

Table S7. Distributions of nests of *Dipogon sperconsus* in bundles per year during 2013-2016.

Year: 2013<br>Site	Bundle number	No. used canes	No. unused canes	No. total canes	
A	1	6	9	15	
	2	2	13	15	
	3	0	15	15	
	4	2	13	15	
	5	2	13	15	
	6	1	14	15	
	7	6	9	15	
	8	0	15	15	
	9	3	12	15	
	10	4	11	15	
	11	3	12	15	
	12	2	13	15	
	13	6	9	15	
	14	5	10	15	
	15	0	15	15	
	16	1	14	15	
B	1	4	11	15	
	2	13	2	15	
	3	5	10	15	
	4	10	5	15	
	5	2	13	15	
	6	1	14	15	
	7	1	14	15	
	8	1	14	15	
	9	3	12	15	
	10	2	13	15	
	11	1	14	15	
	12	3	12	15	
	13	3	12	15	
	14	0	15	15	
	15	1	14	15	q
	16	3	12	15	
Total	32	96	384	480	OK
	#data=	p=	q=		
	32	0.2	0.8	1	OK
	Mean	3	12	15	OK
	Variace	8.258065	8.25806452		OK
	Max used	13			

Year: 2014	Bundle number	No. used canes	No. unused canes	No. total canes	
Site					
C	1	1	14	15	
	2	0	15	15	
	3	1	14	15	
	4	1	14	15	
	5	3	12	15	
	6	2	13	15	
	7	1	14	15	
	8	3	12	15	
	9	3	12	15	
	10	4	11	15	
	11	2	13	15	
	12	3	12	15	
	13	1	14	15	
	14	5	10	15	
	15	1	14	15	
	16	1	14	15	
D	1	3	12	15	
	2	2	13	15	
	3	1	14	15	
	4	1	14	15	
	5	2	13	15	
	6	4	11	15	
	7	8	7	15	
	8	0	15	15	
	9	2	13	15	
	10	0	15	15	
	11	0	15	15	
	12	5	10	15	
	13	3	12	15	
	14	0	15	15	
	15	0	15	15	
	16	0	15	15	
Total	32	63	417	480	OK
	#data=	p=	q=		
	32	0.13125	0.86875	1	OK
	Mean	1.96875	13.03125	15	OK
	Variace	3.3860887	3.3860887		OK
	Max used	8			

Year: 2015<br>Site	Bundle number	No. used canes	No. unused canes	No. total canes	
E	1	0	15	15	
	2	0	15	15	
	3	0	15	15	
	4	1	14	15	
	5	1	14	15	
	6	15	0	15	
	7	1	14	15	
	8	11	4	15	
	9	4	11	15	
	10	3	12	15	
	11	3	12	15	
	12	3	12	15	
	13	2	13	15	
	14	2	13	15	
	15	4	11	15	
	16	3	12	15	
F	1	0	15	15	
	2	0	15	15	
	3	4	11	15	
	4	1	14	15	
	5	0	15	15	
	6	3	12	15	
	7	0	15	15	
	8	2	13	15	
	9	0	15	15	
	10	4	11	15	
	11	11	4	15	
	12	4	11	15	
	13	1	14	15	
	14	2	13	15	
	15	3	12	15	
G	1	0	15	15	
	2	0	15	15	
	3	0	15	15	
	4	0	15	15	
	5	3	12	15	
	6	1	14	15	
	7	0	15	15	
	8	0	15	15	
	9	0	15	15	
	10	4	11	15	
	11	3	12	15	
	12	2	13	15	
	13	6	9	15	
	14	1	14	15	
	15	0	15	15	
	16	2	13	15	
H	1	0	15	15	
	2	10	5	15	
	3	0	15	15	
	4	2	13	15	
	5	0	15	15	
	6	3	12	15	
	7	1	14	15	
	8	5	10	15	
	9	0	15	15	
	10	3	12	15	
	11	0	15	15	
	12	0	15	15	
	13	2	13	15	
	14	4	11	15	
	15	0	15	15	
	16	0	15	15	
	17	1	14	15	
	18	0	15	15	
	19	0	15	15	
	20	1	14	15	
I	1	0	15	15	
	2	0	15	15	
	3	1	14	15	
Total	70	143	907	1050	OK
	#data=	p=	q=		
	70	0.1361905	0.8638095	1	OK
	Mean	2.0428571	12.957143	15	OK
	Variance	8.4184265	8.4184265		OK
	Max used	15			

Year: 2016 Site	Bundle number	No. used canes	No. unused canes	No. total canes	
J	1	6	9	15	
	2	9	6	15	
	3	2	13	15	
	4	6	9	15	
	5	8	7	15	
	6	0	15	15	
	7	0	15	15	
	8	0	15	15	
	9	0	15	15	
	10	1	14	15	
	11	0	15	15	
	12	3	12	15	
	13	0	15	15	
	14	0	15	15	
	15	2	13	15	
	16	1	14	15	
K	1	2	13	15	
	2	0	15	15	
	3	0	15	15	
	4	1	14	15	
	5	2	13	15	
	6	1	14	15	
	7	0	15	15	
	8	0	15	15	
	9	0	15	15	
	10	0	15	15	
	11	3	12	15	
	12	1	14	15	
	13	0	15	15	
	14	0	15	15	
	15	1	14	15	
	16	0	15	15	
L	1	0	15	15	
	2	1	14	15	
	3	0	15	15	
	4	2	13	15	
	5	2	13	15	
	6	4	11	15	
	7	0	15	15	
	8	3	12	15	
	9	2	13	15	
	10	0	15	15	
	11	0	15	15	
	12	3	12	15	
	13	0	15	15	
	14	0	15	15	
	15	0	15	15	
	16	3	12	15	
	17	1	14	15	
	18	6	9	15	
	19	3	12	15	
	20	3	12	15	
	21	3	12	15	
M	1	0	15	15	
	2	5	10	15	
	3	4	11	15	
	4	2	13	15	
	5	5	10	15	
	6	1	14	15	
	7	0	15	15	
	8	1	14	15	
	9	1	14	15	
	10	1	14	15	
	11	2	13	15	
	12	0	15	15	
	13	0	15	15	
	14	2	13	15	
	15	1	14	15	
	16	0	15	15	
Total	69	110	925	1035	OK
	#data=	p=	q=		
	69	0.1062802	0.8937198	1	OK
	Mean	1.53125	13.46875	15	OK
	Variace	4.1858483	4.1858483		OK
	Max used	8			

Suppementary Table S8. Yearly observed and expected distributions of nests of *Dipogon sperconsus* per bundle during 2013-2016. The expected distributions are binomial distributions with the same mean assuming random for the used canes.

(1) Year 2013

No.nests	Observed frequency	Expected frequency	χ^2 test		
			χ^2 value	9.984870138	
0	4	1.125899907	df	2	
1	7	4.222124651	P	0.006789112	P<0.01 Not supported: not random
2	6	7.388718139	Obs. Mean	3	
3	6	8.00444465	Obs. Var. (OV)	8.258064516	
4	2	6.003333488	Exp. Mean	3	np
5	2	3.301833418	Exp. Var. (EV)	2.4	npq
6	3	1.375763924	OV/EV	3.440860215	Binomial not supported
7	0	0.442209833			strong trend for aggregated distribution
8	0	0.110552458			
9	0	0.021496311			
10	1	0.003224447			
11	0	0.000366414			
12	0	3.05345E-05			
13	1	1.76161E-06			
14	0	6.29146E-08			
15	0	1.04858E-09			

(2) Year 2014

No.nests	Observed frequency	Expected frequency	χ^2 test		
			χ2 value	n/a	Observed freq. (>5.0) ≤ 3
0	7	3.877682461	df	n/a	χ^2 test: not applicable
1	9	8.787553778	P	n/a	
2	5	9.293312269	Obs. Mean	1.96875	
3	6	6.08411091	Obs. Var. (OV)	3.38608871	
4	2	2.757546671	Exp. Mean	1.96875	np
5	2	0.916537095	Exp. Var. (EV)	1.710351563	npq
6	0	0.230782722	OV/EV	1.979761813	Binomial not supported
7	0	0.044828298			trend for aggregated distribution
8	1	0.006772621			
9	0	0.000795824			
10	0	7.21394E-05			
11	0	4.95398E-06			
12	0	2.49481E-07			
13	0	8.69801E-09			
14	0	1.87727E-10			
15	0	1.89077E-12			

(3) Year 2015

No.nests	Observed frequency	Expected frequency	χ^2 test		
			χ^2 value	65.2294499	
0	28	7.786958495	df	2	
1	11	18.41568464	P	6.84865E-15	P<0.001 Not supported: not random
2	8	20.32425615	Obs. Mean	2.042857143	
3	10	13.88562741	Obs. Var. (OV)	8.418426501	
4	7	6.567733361	Exp. Mean	2.042857143	np
5	1	2.278069367	Exp. Var. (EV)	1.764639456	npq
6	1	0.598610657	OV/EV	4.770621258	Binomial not supported
7	0	0.12134382			aggregated distribution
8	0	0.019131385			
9	0	0.002346015			
10	1	0.000221927			
11	2	1.59044E-05			
12	0	8.35842E-07			
13	0	3.0411E-08			
14	0	6.84954E-10			
15	1	7.19945E-12			

(4) Year 2016

No.nests	Observed frequency	Expected frequency	χ^2 test		
			χ^2 value	29.49167607	
0	29	12.7899791	df	1	
1	13	22.81455732	P	5.61574E-08	P<0.0001 Not supported: not random
2	10	18.99157744	Obs. Mean	0.106280193	
3	8	9.786650719	Obs. Var. (OV)	4.185848252	
4	2	3.49145377	Exp. Mean	0.106280193	np
5	2	0.913439797	Exp. Var. (EV)	1.424770706	npq
6	3	0.181042122	OV/EV	2.937910103	Binomial not supported
7	0	0.027680572			aggregated distribution
8	1	0.003291744			
9	1	0.000304462			
10	0	2.17237E-05			
11	0	1.17426E-06			
12	0	4.65471E-08			
13	0	1.27738E-09			
14	0	2.17007E-11			
15	0	1.72042E-13			

Supplementary Table S9. Rearing records of *Dipogon sperconsus* and unknown *D.* spp. in 2013.

Nest-withdrawing date	Species	Site	No. of cell	Sex: f, female; m, male	Developmental stage at nest-withdrawing: Eg, egg; La, larva; Co, cocoon	Estimated oviposition day*	Emergence day	Duration from oviposition to emergence
2013/6/17	*D. sperconsus*	A	1-15-1	f	La	2013/6/14	2013/7/10	26
2013/6/17	*D. sperconsus*	A	1-15-3	f	La	2013/6/16	2013/7/11	25
2013/6/17	*D. sperconsus*	A	1-15-4	f	La	2013/6/17	2013/7/10	23
2013/6/17	*D. sperconsus*	A	4-14-2	f	Eg	2013/6/17	2013/7/17	30
2013/6/17	*D. sperconsus*	A	1-15-2	-	La	2013/6/14	-	-
2013/6/17	*D. sperconsus*	A	4-14-1	-	Eg	2013/6/16	-	-
2013/6/17	*D. sperconsus*	A	4-14-3	-	Eg	2013/6/16	-	-
2013/7/2	*D. sperconsus*	A	13-12-1	m	La	2013/6/29	2013/7/20	21
2013/7/2	*D. sperconsus*	A	13-12-2	f	La	2013/6/28	2013/7/21	23
2013/7/2	*D. sperconsus*	A	13-12-3	m	La	2013/6/30	2013/7/21	21
2013/7/2	*D. sperconsus*	A	13-12-4	f	La	2013/6/30	2013/7/22	22
2013/7/2	*D. sperconsus*	A	13-12-5	f	La	2013/6/30	2013/7/24	24
2013/7/2	*D. sperconsus*	B	1-12-2	f	Eg	2013/7/1	2013/7/24	23
2013/7/2	*D. sperconsus*	B	1-15-1	m	Eg	2013/7/1	2013/7/23	22
2013/7/2	*D. sperconsus*	B	1-15-3	f	Eg	2013/7/1	2013/7/24	23
2013/7/2	*D. sperconsus*	B	2-2-2	m	La	2013/7/1	2013/7/24	23
2013/7/2	*D. sperconsus*	B	2-2-4	f	Eg	2013/7/1	2013/7/23	22
2013/7/2	*D. sperconsus*	B	2-2-5	f	Eg	2013/7/1	2013/7/25	24
2013/7/2	*D.* spp.	A	12-9-1	-	Eg	2013/6/30	-	-
2013/7/2	*D.* spp.	A	12-9-3	-	Eg	2013/6/30	-	-
2013/7/2	*D.* spp.	A	13-13-1	-	Eg	2013/6/30	-	-
2013/7/2	*D.* spp.	A	13-13-2	-	Eg	2013/6/30	-	-
2013/7/2	*D.* spp.	A	13-13-3	-	Eg	2013/6/30	-	-
2013/7/2	*D. sperconsus*	A	13-12-6	-	Eg	2013/6/30	-	-
2013/7/2	*D. sperconsus*	A	13-12-7	-	Eg	2013/6/30	-	-
2013/7/2	*D. sperconsus*	B	1-12-1	-	Eg	2013/6/30	-	-
2013/7/2	*D. sperconsus*	B	1-12-3	-	Eg	2013/6/30	-	-
2013/7/2	*D. sperconsus*	B	1-15-2	-	Eg	2013/6/30	-	-
2013/7/2	*D. sperconsus*	B	1-15-4	-	Eg	2013/6/30	-	-
2013/7/2	*D. sperconsus*	B	2-2-3	-	Eg	2013/6/30	-	-
2013/7/2	*D. sperconsus*	B	2-2-6	-	Eg	2013/6/30	-	-
2013/7/8	*D. sperconsus*	B	2-4-1	m	La	2013/7/4	2013/7/25	21
2013/7/8	*D. sperconsus*	B	2-4-2	f	La	2013/7/4	2013/7/25	21
2013/7/8	*D. sperconsus*	B	2-4-3	f	La	2013/7/10	2013/7/30	20
2013/7/8	*D.* spp.	A	12-6-1	-	La	2013/7/3	-	-
2013/7/8	*D.* spp.	B	13-11-1	-	Eg	2013/7/6	-	-
2013/7/8	*D.* spp.	B	13-11-2	-	Eg	2013/7/6	-	-
2013/7/17	*D. sperconsus*	A	1-2-1	f	Co	2013/7/8	2013/7/31	23
2013/7/17	*D. sperconsus*	A	1-2-2	f	Co	2013/7/9	2013/8/1	23
2013/7/17	*D. sperconsus*	B	1-15-1	f	Co	2013/7/9	2013/8/1	23
2013/7/17	*D. sperconsus*	B	2-9-4	f	La	2013/7/10	2013/8/2	23
2013/7/25	*D.* spp.	A	1-14-2	-	Eg	2013/7/23	-	-
2013/7/25	*D.* spp.	A	1-14-3	-	Eg	2013/7/23	-	-
2013/7/25	*D.* spp.	A	1-14-4	-	Eg	2013/7/23	-	-
2013/7/25	*D.* spp.	B	2-12-1	-	La	2013/7/24	-	-
2013/7/25	*D.* spp.	B	2-12-2	-	La	2013/7/22	-	-
2013/7/25	*D.* spp.	B	2-12-3	-	Eg	2013/7/23	-	-
2013/8/1	*D. sperconsus*	A	1-15-2	m	Co	2013/7/24	2013/8/14	21
2013/8/1	*D. sperconsus*	A	5-15-2	f	La	2013/7/26	2013/8/14	19
2013/8/1	*D. sperconsus*	A	5-15-3	f	La	2013/7/26	2013/8/16	21
2013/8/1	*D. sperconsus*	A	5-15-4	f	La	2013/7/26	2013/8/16	21
2013/8/1	*D. sperconsus*	A	5-15-5	m	La	2013/7/28	2013/8/14	17
2013/8/1	*D. sperconsus*	A	5-15-6	f	La	2013/7/27	2013/8/16	20
2013/8/1	*D. sperconsus*	B	4-6-1	m	La	2013/7/30	2013/8/13	14
2013/8/1	*D. sperconsus*	B	4-6-2	m	La	2013/7/27	2013/8/13	17
2013/8/1	*D.* spp.	A	2-1-1	-	Eg	2013/7/30	-	-
2013/8/1	*D.* spp.	A	2-3-1	-	Eg	2013/7/30	-	-
2013/8/1	*D.* spp.	A	16-1-1	-	Eg	2013/7/30	-	-
2013/8/1	*D. sperconsus*	A	5-15-1	m	La	2013/7/26	-	-
2013/8/7	*D. sperconsus*	A	1-9-1	m	La	2013/8/2	2014/5/6	277
2013/8/7	*D. sperconsus*	A	7-3-1	f	La	2013/8/1	2013/8/24	23
2013/8/7	*D. sperconsus*	A	7-3-2	m	La	2013/8/3	2013/8/23	20
2013/8/7	*D. sperconsus*	A	7-3-3	f	La	2013/8/2	2013/8/26	24
2013/8/7	*D. sperconsus*	A	7-3-4	m	La	2013/8/3	2013/8/24	21
2013/8/7	*D. sperconsus*	A	7-6-1	f	La	2013/8/1	2013/8/22	21
2013/8/7	*D. sperconsus*	A	7-6-2	m	La	2013/8/2	2013/8/21	19
2013/8/7	*D. sperconsus*	B	2-15-1	m	La	2013/8/2	2014/5/13	284
2013/8/7	*D. sperconsus*	B	2-15-2	f	La	2013/8/1	2014/5/13	285
2013/8/7	*D. sperconsus*	B	9-9-1	m	La	2013/8/2	2013/8/23	21
2013/8/7	*D. sperconsus*	B	9-9-2	f	La	2013/8/3	2013/8/27	24
2013/8/7	*D. sperconsus*	B	11-10-1	m	La	2013/8/2	2014/5/8	279
2013/8/7	*D. sperconsus*	B	11-10-2	f	La	2013/8/1	2014/5/13	285
2013/8/7	*D.* spp.	A	7-5-1	-	Eg	2013/8/5	-	-
2013/8/7	*D.* spp.	A	11-7-1	-	Eg	2013/8/5	-	-
2013/8/7	*D.* spp.	A	11-7-2	-	Eg	2013/8/5	-	-
2013/8/7	*D.* spp.	B	2-12-1	-	Eg	2013/8/5	-	-
2013/8/7	*D.* spp.	B	2-12-2	-	Eg	2013/8/5	-	-
2013/8/7	*D.* spp.	B	4-15-2	-	Eg	2013/8/5	-	-
2013/8/12	*D. sperconsus*	A	1-12-1	m	La	2013/8/7	2014/5/10	276
2013/8/12	*D. sperconsus*	A	1-12-3	m	La	2013/8/8	2014/5/10	275
2013/8/12	*D. sperconsus*	A	10-15-1	m	La	2013/8/8	2014/5/10	275
2013/8/12	*D. sperconsus*	A	14-12-2	f	La	2013/8/7	2014/5/13	279
2013/8/12	*D. sperconsus*	B	2-3-1	m	La	2013/8/7	2014/5/12	278
2013/8/12	*D. sperconsus*	B	2-3-2	f	La	2013/8/7	2014/5/13	279
2013/8/12	*D. sperconsus*	B	4-6-1	m	La	2013/8/9	2013/8/26	17
2013/8/12	*D. sperconsus*	B	4-6-3	f	La	2013/8/6	2014/5/13	280
2013/8/12	*D.* spp.	B	2-15-1	-	La	2013/8/8	-	-
2013/8/12	*D.* spp.	B	5-1-1	-	Eg	2013/8/10	-	-

2013/8/12	*D.* spp.	B	10-9-1	-	La	2013/8/7	-	-
2013/8/12	*D. sperconsus*	A	1-12-2	-	La	2013/8/8	-	-
2013/8/12	*D. sperconsus*	A	10-15-2	-	Eg	2013/8/10	-	-
2013/8/12	*D. sperconsus*	B	2-3-3	-	La	2013/8/8	-	-
2013/8/12	*D. sperconsus*	B	4-6-2	-	La	2013/8/7	-	-
2013/8/15	*D.* spp.	A	14-9-4	-	Eg	2013/8/13	-	-
2013/8/15	*D.* spp.	B	3-12-2	-	Eg	2013/8/13	-	-
2013/8/19	*D. sperconsus*	B	3-12-1	f	La	2013/8/15	2014/5/13	271
2013/8/19	*D. sperconsus*	B	3-12-2	m	La	2013/8/16	2014/5/12	269
2013/8/23	*D.* spp.	B	4-12-1	-	Eg	2013/8/21	-	-
2013/8/23	*D.* spp.	B	4-12-2	-	Eg	2013/8/21	-	-
2013/8/27	*D.* spp.	A	14-14-1	-	Eg	2013/8/25	-	-
2013/9/4	*D. sperconsus*	A	9-12-1	f	La	2013/8/30	2014/5/16	259
2013/9/4	*D. sperconsus*	A	13-9-1	m	La	2013/8/29	2014/5/12	256
2013/9/4	*D. sperconsus*	A	13-9-2	f	La	2013/8/30	2014/5/13	256
2013/9/4	*D. sperconsus*	A	13-9-3	m	La	2013/9/1	2014/5/10	251
2013/9/4	*D. sperconsus*	A	13-9-4	f	La	2013/9/2	2014/5/13	253
2013/9/4	*D. sperconsus*	A	14-3-1	f	La	2013/8/27	2014/5/13	259
2013/9/4	*D. sperconsus*	A	14-3-2	m	La	2013/8/28	2014/5/13	258
2013/9/4	*D. sperconsus*	A	14-3-3	f	La	2013/8/28	2014/5/13	258
2013/9/4	*D. sperconsus*	A	14-3-5	m	La	2013/8/31	2014/5/13	255
2013/9/4	*D. sperconsus*	B	4-12-1	f	La	2013/8/30	2014/5/18	261
2013/9/4	*D.* spp.	A	13-1-1	-	La	2013/8/29	-	-
2013/9/4	*D.* spp.	A	13-1-2	-	La	2013/8/30	-	-
2013/9/4	*D.* spp.	A	13-1-3	-	La	2013/9/1	-	-
2013/9/4	*D.* spp.	B	2-12-3	-	Eg	2013/9/2	-	-
2013/9/4	*D.* spp.	B	2-12-4	-	Eg	2013/9/2	-	-
2013/9/4	*D.* spp.	B	2-12-5	-	Eg	2013/9/2	-	-
2013/9/4	*D.* spp.	B	3-15-1	-	Eg	2013/9/2	-	-
2013/9/4	*D.* spp.	B	3-15-2	-	Eg	2013/9/2	-	-
2013/9/4	*D.* spp.	B	10-3-1	-	Eg	2013/9/2	-	-
2013/9/19	*D. sperconsus*	A	6-15-1	m	Co	2013/9/9	2014/5/13	246
2013/9/19	*D. sperconsus*	A	7-15-1	f	La	2013/9/15	2014/5/13	240
2013/9/19	*D. sperconsus*	A	7-15-3	m	La	2013/9/9	2014/5/13	246
2013/9/19	*D. sperconsus*	A	9-8-2	f	La	2013/9/15	2014/5/12	239
2013/9/19	*D. sperconsus*	A	10-12-2	m	Co	2013/9/9	2014/5/13	246
2013/9/19	*D. sperconsus*	A	11-12-1	f	Co	2013/9/11	2014/5/15	246
2013/9/19	*D. sperconsus*	A	13-12-1	f	Co	2013/9/9	2014/5/13	246
2013/9/19	*D. sperconsus*	A	13-12-5	f	La	2013/9/12	2014/5/13	243
2013/9/19	*D. sperconsus*	A	13-12-6	f	La	2013/9/13	2014/5/16	245
2013/9/19	*D. sperconsus*	B	3-15-1	m	Co	2013/9/6	2014/5/10	246
2013/9/19	*D. sperconsus*	B	3-15-2	f	Co	2013/9/9	2014/5/13	246
2013/9/19	*D. sperconsus*	B	3-15-3	m	Co	2013/9/8	2014/5/12	246
2013/9/19	*D. sperconsus*	B	3-15-4	f	La	2013/9/12	2014/5/13	243
2013/9/19	*D. sperconsus*	B	3-15-5	m	La	2013/9/13	2014/5/12	241
2013/9/19	*D. sperconsus*	B	4-5-1	f	La	2013/9/11	2014/5/13	244
2013/9/19	*D. sperconsus*	B	4-9-3	f	La	2013/9/12	2014/5/16	246
2013/9/19	*D. sperconsus*	B	4-9-4	m	La	2013/9/16	2014/5/13	239
2013/9/19	*D. sperconsus*	B	4-15-1	f	La	2013/9/12	2014/5/16	246
2013/9/19	*D. sperconsus*	B	13-15-1	m	Co	2013/9/8	2014/5/12	246
2013/9/19	*D. sperconsus*	B	16-15-3	f	La	2013/9/12	2014/5/13	243
2013/9/19	*D. sperconsus*	B	16-15-4	f	La	2013/9/12	2014/5/13	243
2013/9/19	*D. sperconsus*	B	16-15-5	m	La	2013/9/14	2014/5/12	240
2013/9/19	*D.* spp.	A	10-9-1	-	La	2013/9/18	-	-
2013/9/19	*D.* spp.	B	1-3-1	-	Eg	2013/9/17	-	-
2013/9/19	*D.* spp.	B	3-14-2	-	La	2013/9/16	-	-
2013/9/19	*D.* spp.	B	12-8-2	-	La	2013/9/18	-	-
2013/9/19	*D.* spp.	B	13-6-2	-	La	2013/9/16	-	-
2013/9/19	*D. sperconsus*	A	7-15-4	-	Eg	2013/9/17	-	-
2013/9/19	*D. sperconsus*	A	9-8-3	-	La	2013/9/12	-	-
2013/9/19	*D. sperconsus*	A	10-12-4	-	La	2013/9/17	-	-
2013/9/19	*D. sperconsus*	A	13-12-4	-	La	2013/9/16	-	-
2013/9/19	*D. sperconsus*	B	4-9-1	-	La	2013/9/16	-	-
2013/9/19	*D. sperconsus*	B	4-9-2	-	La	2013/9/16	-	-
2013/9/19	*D. sperconsus*	B	13-15-2	-	La	2013/9/16	-	-
2013/9/19	*D. sperconsus*	B	16-15-6	-	La	2013/9/16	-	-
2013/9/25	*D. sperconsus*	B	5-11-1	f	Co	2013/9/12	2014/5/16	246
2013/9/25	*D. sperconsus*	B	5-11-2	m	Co	2013/9/9	2014/5/13	246

Summer generations: 21.7±2.8 (n=39)

Overwintering generation: 256.6±15.1 (n=48)

*The oviposition day was estimated as follows: when we first detected an egg in a cell on a certain day in the daily survey, we identified its oviposition day as the previous day; when we found a larva in a collected nest and recorded the spinning day of the larva, we estimated its oviposition day by counting back to include the total average time of the egg and larval periods from that day.

Supplementary Table S10. Rearing records of *Dipogon sperconsus* and unknown *D*. spp. in 2014.

Nest-withdrawing date	Species	Site	No. of cell	Sex: f, female; m, male	Developmental stage at nest-withdrawing: Eg, egg; La, larva; Co, cocoon	Estimated oviposition day*	Emergence day	Duration from oviposition to emergence
2014/7/1	*D. sperconsus*	C	5-12-1	m	La	2014/7/2	2014/7/23	21
2014/7/1	*D. sperconsus*	C	5-12-2	f	La	2014/7/1	2014/7/24	23
2014/7/1	*D. sperconsus*	C	5-14-1	f	Eg	2014/7/3	2014/7/26	23
2014/7/1	*D. sperconsus*	D	1-4-1	m	La	2014/7/2	2014/7/23	21
2014/7/1	*D. sperconsus*	D	12-14-1	f	Eg	2014/7/1	2014/7/24	23
2014/7/1	*D. sperconsus*	D	12-14-2	m	Eg	2014/7/4	2014/7/25	21
2014/7/7	*D. sperconsus*	D	4-12-1	f	La	2014/7/4	2014/7/27	23
2014/7/7	*D. sperconsus*	D	7-12-1	f	La	2014/7/5	2014/7/28	23
2014/7/7	*D. sperconsus*	D	7-12-2	m	La	2014/7/6	2014/7/26	20
2014/7/7	*D. sperconsus*	D	7-12-3	-	Eg	2014/7/7	-	-
2014/7/7	*D. sperconsus*	D	7-12-4	-	Eg	2014/7/7	-	-
2014/7/7	*D. sperconsus*	D	7-12-5	-	Eg	2014/7/7	-	-
2014/7/8	*D. sperconsus*	D	7-15-1	m	Eg	2014/7/7	2014/7/31	24
2014/7/8	*D. sperconsus*	D	7-15-2	-	Eg	2014/7/7	-	-
2014/7/8	*D. sperconsus*	D	7-15-3	-	Eg	2014/7/7	-	-
2014/7/14	*D. sperconsus*	C	6-15-2	m	Eg	2014/7/14	2014/8/4	21
2014/7/14	*D. sperconsus*	D	7-12-3	f	Eg	2014/7/13	2014/7/31	18
2014/7/14	*D. sperconsus*	C	6-15-1	-	Eg	2014/7/12	-	-
2014/7/14	*D. sperconsus*	D	7-12-2	-	Eg	2014/7/14	-	-
2014/7/15	*D. sperconsus*	D	7-12-1	f	Eg	2014/7/14	2014/8/6	23
2014/7/15	*D. sperconsus*	D	7-12-2	-	Eg	2014/7/14	-	-
2014/7/16	*D. sperconsus*	D	7-12-2	m	Eg	2014/7/15	2014/8/6	22
2014/7/16	*D. sperconsus*	D	7-12-1	-	Eg	2014/7/15	-	-
2014/7/21	*D. sperconsus*	D	9-12-1	m	La	2014/7/16	2014/8/6	21
2014/7/21	*D. sperconsus*	D	9-12-3	m	La	2014/7/19	2014/8/8	20
2014/7/21	*D.* spp.	C	13-12-1	-	Eg	2014/7/19	-	-
2014/7/21	*D.* spp.	C	13-12-2	-	Eg	2014/7/22	-	-
2014/7/28	*D. sperconsus*	D	7-9-3	-	Eg	2014/7/28	-	-
2014/7/28	*D. sperconsus*	D	7-9-4	-	Eg	2014/7/28	-	-
2014/7/29	*D.* spp.	D	6-8-1	-	Eg	2014/7/28	-	-
2014/8/1	*D. sperconsus*	D	6-9-1	f	Eg	2014/8/1	2014/8/23	22
2014/8/4	*D.* spp.	C	9-2-1	-	La	2014/7/29	-	-
2014/8/11	*D.* spp.	C	14-12-1	-	Eg	2014/8/12	-	-
2014/8/12	*D. sperconsus*	C	14-12-1	m	Eg	2014/8/11	2015/4/25	257
2014/8/14	*D.* spp.	C	14-12-1	-	Eg	2014/8/15	-	-
2014/8/25	*D. sperconsus*	C	12-12-1	m	La	2014/8/22	2015/4/26	247
2014/8/25	*D. sperconsus*	C	12-12-2	f	La	2014/8/21	2015/4/27	249
2014/8/26	*D. sperconsus*	C	12-12-1	f	Eg	2014/8/25	2015/4/28	246
2014/8/27	*D.* spp.	C	8-12-1	-	Eg	2014/8/26	-	-
2014/9/3	*D. sperconsus*	C	9-15-1	f	La	2014/8/31	2015/4/27	239
2014/9/3	*D. sperconsus*	C	9-15-2	m	La	2014/8/31	2015/4/26	238
2014/9/3	*D. sperconsus*	C	12-12-1	f	Eg	2014/9/2	2015/4/27	237
2014/9/3	*D.* spp.	C	4-15-1	-	La	2014/8/31	-	-
2014/9/4	*D.* spp.	C	9-12-1	-	Eg	2014/9/3	-	-
2014/9/8	*D. sperconsus*	C	3-12-2	f	La	2014/9/5	2015/4/28	235
2014/9/8	*D. sperconsus*	C	10-15-1	m	Eg	2014/9/6	2015/4/26	232
2014/9/8	*D.* spp.	C	11-6-2	-	Eg	2014/9/9	-	-
2014/9/8	*D. sperconsus*	C	3-12-4	-	Eg	2014/9/8	-	-
2014/9/9	*D. sperconsus*	C	10-15-1	f	Eg	2014/9/8	2015/4/27	231
2014/9/12	*D. sperconsus*	C	10-8-1	f	Eg	2014/9/10	2015/4/27	229
2014/9/12	*D. sperconsus*	D	12-12-1	f	La	2014/9/9	2015/4/29	232
2014/9/12	*D. sperconsus*	D	12-12-2	m	Eg	2014/9/10	2015/4/26	228
2014/9/12	*D. sperconsus*	D	12-12-3	-	Eg	2014/9/13	-	-
2014/9/15	*D. sperconsus*	C	10-15-1	m	Eg	2014/9/14	2015/4/25	223
2014/9/15	*D. sperconsus*	D	13-15-1	f	Eg	2014/9/14	2015/5/1	229
2014/9/15	*D.* spp.	D	12-3-1	-	Eg	2014/9/13	-	-
2014/9/15	*D.* spp.	D	12-3-2	-	Eg	2014/9/16	-	-
2014/9/15	*D.* spp.	D	12-3-3	-	Eg	2014/9/13	-	-
2014/9/15	*D. sperconsus*	C	10-15-2	-	Eg	2014/9/16	-	-
2014/9/15	*D. sperconsus*	C	10-15-3	-	Eg	2014/9/13	-	-
2014/9/18	*D. sperconsus*	C	11-9-1	f	Eg	2014/9/17	2015/4/27	222
2014/9/18	*D. sperconsus*	D	2-15-1	f	Eg	2014/9/17	2015/4/28	223
2014/9/18	*D. sperconsus*	D	5-12-1	m	La	2014/9/18	2015/4/26	220
2014/9/18	*D. sperconsus*	D	13-9-1	f	Eg	2014/9/16	2015/4/30	226
2014/9/18	*D.* spp.	C	16-14-2	-	Eg	2014/9/19	-	-
2014/9/18	*D.* spp.	D	9-15-1	-	Eg	2014/9/19	-	-
2014/9/18	*D.* spp.	D	12-15-1	-	Eg	2014/9/19	-	-
2014/9/18	*D.* spp.	D	12-15-2	-	Eg	2014/9/21	-	-
2014/9/18	*D. sperconsus*	D	13-9-2	-	Eg	2014/9/16	-	-
2014/9/18	*D. sperconsus*	D	13-9-3	-	Eg	2014/9/19	-	-
2014/9/22	*D. sperconsus*	D	2-15-1	f	Eg	2014/9/21	2015/4/29	220
2014/9/22	*D.* spp.	D	1-15-1	-	Eg	2014/9/20	-	-
2014/9/22	*D.* spp.	D	13-6-1	-	Eg	2014/9/20	-	-
2014/9/26	*D. sperconsus*	D	1-9-2	f	Eg	2014/9/25	2015/4/28	215
2014/9/26	*D.* spp.	C	15-11-2	-	Eg	2014/9/26	-	-
2014/9/26	*D.* spp.	C	15-11-3	-	Eg	2014/9/24	-	-
2014/9/26	*D. sperconsus*	D	1-9-1	-	Eg	2014/9/27	-	-
						Summer generations: 21.7±1.5 (n=17)		
						Overwinter generation: 232.3±10.9 (n=21)		

*The oviposition day was estimated as follows: when we first detected an egg in a cell on a certain day in the daily survey, we identified its oviposition day as the previous day; when we found a larva in a collected nest and recorded the spinning day of the larva, we estimated its oviposition day by counting back to include the total average time of the egg and larval periods from that day.

Supplementary Table S11. Rearing records of *Dipogon sperconsus* and unknown *D*. spp. in 2015.

Nest-withdrawing date	Species	Site	No. of cell	Sex: f, female; m, male	Developmental stage at nest-withdrawing: Eg, egg; La, larva; Co, cocoon	Estimated oviposition day*	Emergence day	Duration from oviposition to emergence
2015/6/22	*D.* spp.	E	4-5-1	-	Co	2015/6/23	-	-
2015/6/22	*D.* spp.	E	4-5-2	-	Co	2015/6/23	-	-
2015/6/22	*D.* spp.	E	4-5-3	-	Co	2015/6/23	-	-
2015/6/22	*D.* spp.	E	4-5-4	-	La	2015/6/15	-	-
2015/6/22	*D.* spp.	E	11-15-1	-	La	2015/6/26	-	-
2015/6/22	*D.* spp.	F	3-6-1	-	Eg	2015/6/20	-	-
2015/6/22	*D.* spp.	F	3-6-2	-	Eg	2015/6/20	-	-
2015/6/22	*D.* spp.	F	3-6-3	-	Eg	2015/6/20	-	-
2015/6/22	*D.* spp.	F	3-6-4	-	Eg	2015/6/20	-	-
2015/7/4	*D. sperconsus*	F	4-6-1	m	La	2015/6/30	2015/7/21	21
2015/7/4	*D. sperconsus*	F	10-14-1	m	La	2015/7/3	2015/7/23	20
2015/7/4	*D. sperconsus*	F	10-14-2	f	Eg	2015/7/4	2015/7/28	24
2015/7/4	*D. sperconsus*	F	10-14-4	f	Eg	2015/7/4	2015/7/29	25
2015/7/4	*D. sperconsus*	F	11-15-1	f	La	2015/6/28	2015/7/22	24
2015/7/4	*D. sperconsus*	F	11-15-2	m	La	2015/6/29	2015/7/21	22
2015/7/4	*D. sperconsus*	F	11-16-1	f	La	2015/6/29	2015/7/23	24
2015/7/4	*D.* spp.	E	16-3-1	-	Co	2015/6/20	-	-
2015/7/4	*D.* spp.	E	16-3-2	-	La	2015/7/1	-	-
2015/7/4	*D.* spp.	F	11-12-2	-	La	2015/6/29	-	-
2015/7/4	*D. sperconsus*	F	10-14-3	-	Eg	2015/7/3	-	-
2015/7/11	*D. sperconsus*	F	3-12-2	f	La	2015/7/5	2015/7/28	23
2015/7/11	*D.* spp.	E	12-8-1	-	Eg	2015/7/9	-	-
2015/7/11	*D.* spp.	F	3-5-1	-	Co	2015/6/27	-	-
2015/7/11	*D.* spp.	F	3-5-2	-	Co	2015/6/27	-	-
2015/7/11	*D.* spp.	F	10-15-1	-	La	2015/7/6	-	-
2015/7/11	*D.* spp.	F	11-14-1	-	Eg	2015/7/9	-	-
2015/7/11	*D. sperconsus*	F	3-12-1	-	Co	2015/6/27	-	-
2015/7/13	*D.* spp.	F	11-15-1	-	Eg	2015/7/12	-	-
2015/7/14	*D.* spp.	F	13-12-3	-	Eg	2015/7/13	-	-
2015/7/14	*D.* spp.	F	15-15-1	-	Eg	2015/7/13	-	-
2015/7/25	*D. sperconsus*	G	12-12-1	f	La	2015/7/21	2015/8/14	24
2015/7/27	*D. sperconsus*	E	11-15-1	m	Eg	2015/7/26	2015/8/16	21
2015/7/27	*D. sperconsus*	F	11-6-1	f	Eg	2015/7/26	2015/8/19	24
2015/7/27	*D.* spp.	E	13-6-2	-	Eg	2015/7/25	-	-
2015/7/27	*D.* spp.	F	12-12-1	-	Eg	2015/7/25	-	-
2015/7/27	*D.* spp.	F	12-12-2	-	Eg	2015/7/25	-	-
2015/7/27	*D.* spp.	F	12-12-3	-	Eg	2015/7/25	-	-
2015/7/27	*D.* spp.	G	10-9-1	-	Eg	2015/7/25	-	-
2015/7/27	*D.* spp.	G	10-9-2	-	Eg	2015/7/25	-	-
2015/7/27	*D.* spp.	G	12-12-1	-	Eg	2015/7/25	-	-
2015/7/27	*D. sperconsus*	F	11-6-2	-	Eg	2015/7/25	-	-
2015/7/28	*D. sperconsus*	E	13-3-1	m	Eg	2015/7/27	2015/8/16	20
2015/7/28	*D. sperconsus*	F	12-4-1	f	Eg	2015/7/27	2015/8/19	23
2015/7/28	*D.* spp.	F	11-13-1	-	Eg	2015/7/27	-	-
2015/7/28	*D.* spp.	G	11-3-1	-	Eg	2015/7/27	-	-
2015/7/28	*D.* spp.	G	11-3-2	-	Eg	2015/7/27	-	-
2015/7/28	*D.* spp.	G	13-14-1	-	Eg	2015/7/27	-	-
2015/7/28	*D. sperconsus*	F	12-4-2	-	Eg	2015/7/27	-	-
2015/7/29	*D. sperconsus*	E	15-6-2	f	Eg	2015/7/28	2015/8/19	22
2015/7/29	*D. sperconsus*	E	15-6-3	f	Eg	2015/7/28	2015/8/21	24
2015/7/29	*D. sperconsus*	H	2-3-1	f	Eg	2015/7/28	2015/8/21	24
2015/7/29	*D.* spp.	E	6-6-1	-	Eg	2015/7/28	-	-
2015/7/29	*D.* spp.	E	16-12-1	-	Eg	2015/7/28	-	-
2015/7/29	*D. sperconsus*	E	15-6-1	-	Eg	2015/7/28	-	-
2015/7/30	*D. sperconsus*	E	6-2-1	m	Eg	2015/7/29	2015/8/21	23
2015/7/30	*D. sperconsus*	E	6-2-2	f	Eg	2015/7/29	2015/8/21	23
2015/7/30	*D. sperconsus*	F	15-6-2	m	La	2015/7/28	2015/8/22	25
2015/7/30	*D.* spp.	G	11-3-1	-	Eg	2015/7/29	-	-
2015/7/30	*D. sperconsus*	F	15-6-1	-	La	2015/7/28	-	-
2015/7/30	*D. sperconsus*	F	15-6-3	-	La	2015/7/27	-	-
2015/7/30	*D. sperconsus*	F	15-6-4	-	La	2015/7/27	-	-
2015/7/31	*D. sperconsus*	E	11-12-1	f	Eg	2015/7/30	2015/8/22	23
2015/7/31	*D. sperconsus*	F	11-5-3	f	La	2015/7/29	2015/8/22	24
2015/7/31	*D. sperconsus*	F	14-15-3	m	Eg	2015/7/30	2015/8/17	18
2015/7/31	*D. sperconsus*	H	2-12-1	f	Eg	2015/7/30	2015/8/22	23
2015/7/31	*D.* spp.	E	10-3-1	-	Eg	2015/7/30	-	-
2015/7/31	*D.* spp.	E	10-3-2	-	Eg	2015/7/30	-	-
2015/7/31	*D.* spp.	E	10-3-4	-	Eg	2015/7/30	-	-
2015/8/1	*D. sperconsus*	E	9-3-1	f	La	2015/7/29	2015/8/22	24
2015/8/1	*D. sperconsus*	E	9-3-2	m	La	2015/7/30	2015/8/21	22
2015/8/1	*D. sperconsus*	E	10-2-1	f	Eg	2015/7/31	2015/8/22	22
2015/8/1	*D. sperconsus*	F	15-6-1	m	La	2015/8/1	2015/8/21	20
2015/8/1	*D. sperconsus*	H	2-12-1	f	Eg	2015/7/31	2015/8/22	22
2015/8/1	*D.* spp.	E	6-12-2	-	Eg	2015/7/31	-	-
2015/8/1	*D.* spp.	E	9-15-1	-	Eg	2015/7/31	-	-
2015/8/1	*D.* spp.	F	11-14-1	-	La	2015/7/28	-	-
2015/8/1	*D.* spp.	F	11-14-2	-	Eg	2015/7/31	-	-
2015/8/1	*D.* spp.	F	11-14-3	-	Eg	2015/7/31	-	-
2015/8/1	*D.* spp.	G	13-12-3	-	Eg	2015/7/31	-	-
2015/8/1	*D. sperconsus*	E	10-2-2	-	Eg	2015/7/31	-	-
2015/8/1	*D. sperconsus*	F	15-6-2	-	Eg	2015/7/30	-	-
2015/8/2	*D. sperconsus*	G	10-15-2	m	Eg	2015/8/1	2015/8/27	26
2015/8/2	*D.* spp.	E	6-6-2	-	Eg	2015/8/1	-	-
2015/8/2	*D. sperconsus*	G	10-15-1	-	Eg	2015/8/1	-	-
2015/8/3	*D. sperconsus*	E	6-9-1	m	La	2015/7/30	2015/8/21	22
2015/8/3	*D. sperconsus*	E	6-9-2	f	La	2015/7/29	2015/8/23	25
2015/8/3	*D. sperconsus*	E	6-12-2	f	Eg	2015/8/2	2015/8/25	23
2015/8/3	*D. sperconsus*	E	8-6-1	f	La	2015/7/31	2015/8/21	21
2015/8/3	*D. sperconsus*	E	8-6-3	m	Eg	2015/8/2	2015/8/22	20
2015/8/3	*D. sperconsus*	F	6-12-1	f	La	2015/7/28	2015/8/21	24
2015/8/3	*D. sperconsus*	F	6-12-2	f	La	2015/7/28	2015/8/21	24
2015/8/3	*D. sperconsus*	F	6-12-3	m	La	2015/7/29	2015/8/22	24
2015/8/3	*D. sperconsus*	F	6-12-4	f	La	2015/7/31	2015/8/22	22
2015/8/3	*D. sperconsus*	F	6-15-2	f	La	2015/8/2	2015/8/23	21
2015/8/3	*D. sperconsus*	G	14-12-3	f	La	2015/7/31	2015/8/22	22
2015/8/3	*D. sperconsus*	G	16-10-1	f	La	2015/7/28	2015/8/19	22
2015/8/3	*D. sperconsus*	G	16-12-1	f	La	2015/7/28	2015/8/22	25
2015/8/3	*D. sperconsus*	G	16-12-2	m	La	2015/7/29	2015/8/21	23
2015/8/3	*D. sperconsus*	G	16-12-3	f	La	2015/7/30	2015/8/22	23
2015/8/3	*D.* spp.	G	5-8-1	-	Eg	2015/8/2	-	-
2015/8/3	*D. sperconsus*	E	6-9-4	-	La	2015/7/30	-	-
2015/8/3	*D. sperconsus*	E	6-12-1	-	Eg	2015/8/2	-	-
2015/8/3	*D. sperconsus*	E	8-6-4	-	Eg	2015/8/1	-	-

2015/8/3	*D. sperconsus*	G	14-12-1	-	La	2015/8/1	-	-
2015/8/4	*D. sperconsus*	E	8-9-2	f	Eg	2015/8/3	2015/8/26	23
2015/8/4	*D.* spp.	E	6-9-1	-	Eg	2015/8/3	-	-
2015/8/4	*D.* spp.	E	6-9-2	-	Eg	2015/8/3	-	-
2015/8/4	*D.* spp.	G	5-6-1	-	Eg	2015/8/3	-	-
2015/8/4	*D.* spp.	G	5-6-2	-	Eg	2015/8/3	-	-
2015/8/4	*D.* spp.	G	10-8-1	-	Eg	2015/8/3	-	-
2015/8/4	*D.* spp.	G	10-8-2	-	Eg	2015/8/3	-	-
2015/8/4	*D.* spp.	G	10-8-3	-	Eg	2015/8/3	-	-
2015/8/4	*D. sperconsus*	E	8-9-1	-	Eg	2015/8/3	-	-
2015/8/5	*D. sperconsus*	E	6-5-2	m	Eg	2015/8/4	2015/8/27	23
2015/8/5	*D.* spp.	E	8-3-1	-	Eg	2015/8/4	-	-
2015/8/5	*D.* spp.	E	8-3-2	-	Eg	2015/8/4	-	-
2015/8/5	*D.* spp.	F	6-6-1	-	Eg	2015/8/4	-	-
2015/8/5	*D. sperconsus*	E	6-5-1	-	Eg	2015/8/4	-	-
2015/8/6	*D.* spp.	G	5-6-2	-	Eg	2015/8/5	-	-
2015/8/11	*D.* spp.	E	6-8-1	-	La	2015/8/5	-	-
2015/8/11	*D.* spp.	E	8-8-2	-	La	2015/8/5	-	-
2015/8/11	*D.* spp.	F	12-12-1	-	Eg	2015/8/9	-	-
2015/8/19	*D. sperconsus*	E	8-12-1	m	La	2015/8/12	2016/4/21	253
2015/8/19	*D. sperconsus*	E	8-12-2	m	La	2015/8/13	2016/4/20	251
2015/8/19	*D. sperconsus*	G	6-3-2	f	La	2015/8/11	2016/4/22	255
2015/8/19	*D. sperconsus*	H	4-5-1	m	La	2015/8/16	2016/4/21	249
2015/8/19	*D. sperconsus*	H	4-5-2	m	Eg	2015/8/18	2016/4/21	247
2015/8/19	*D.* spp.	F	11-7-1	-	La	2015/8/17	-	-
2015/8/22	*D.* spp.	E	8-3-1	-	Eg	2015/8/20	-	-
2015/8/24	*D. sperconsus*	E	9-14-1	f	Eg	2015/8/22	2016/4/23	245
2015/8/31	*D. sperconsus*	E	6-2-2	f	La	2015/8/27	2016/4/23	240
2015/8/31	*D. sperconsus*	E	9-9-1	f	La	2015/8/25	2016/4/23	242
2015/8/31	*D. sperconsus*	E	9-9-2	m	La	2015/8/26	2016/4/23	241
2015/8/31	*D. sperconsus*	E	12-11-1	f	La	2015/8/25	2016/4/23	242
2015/9/5	*D. sperconsus*	E	5-2-2	f	Eg	2015/9/3	2016/4/24	234
2015/9/5	*D. sperconsus*	H	6-15-1	f	La	2015/8/31	2016/4/24	237
2015/9/6	*D. sperconsus*	E	6-11-1	m	La	2015/9/2	2016/4/22	233
2015/9/6	*D. sperconsus*	H	2-15-1	f	Eg	2015/9/5	2016/4/26	234
2015/9/6	*D. sperconsus*	H	6-15-1	f	Eg	2015/9/5	2016/4/24	232
2015/9/12	*D. sperconsus*	E	14-8-1	m	La	2015/9/8	2016/4/24	229
2015/9/12	*D. sperconsus*	E	14-8-2	m	La	2015/9/8	2016/4/23	228
2015/9/12	*D. sperconsus*	H	2-6-1	f	Eg	2015/9/12	2016/4/25	226
2015/9/12	*D. sperconsus*	H	13-6-1	m	La	2015/9/9	2016/4/23	227
2015/9/12	*D. sperconsus*	H	14-9-2	f	La	2015/9/7	2016/4/23	229
2015/9/12	*D.* spp.	E	6-14-1	-	Eg	2015/9/11	-	-
2015/9/12	*D.* spp.	H	8-6-1	-	Eg	2015/9/11	-	-
2015/9/12	*D.* spp.	H	8-12-1	-	Eg	2015/9/11	-	-
2015/9/12	*D. sperconsus*	E	14-8-3	-	Eg	2015/9/10	-	-
2015/9/12	*D. sperconsus*	H	2-6-2	-	Eg	2015/9/11	-	-
2015/9/13	*D.* spp.	E	14-9-1	-	Eg	2015/9/12	-	-
2015/9/13	*D.* spp.	H	2-9-1	-	Eg	2015/9/12	-	-
2015/9/13	*D.* spp.	H	8-12-1	-	Eg	2015/9/12	-	-
2015/9/13	*D.* spp.	H	14-12-1	-	Eg	2015/9/12	-	-
2015/9/13	*D.* spp.	H	14-12-2	-	Eg	2015/9/12	-	-
2015/9/14	*D.* spp.	H	2-9-1	-	Eg	2015/9/13	-	-
2015/9/14	*D.* spp.	H	8-12-1	-	Eg	2015/9/13	-	-
2015/9/14	*D.* spp.	H	14-6-1	-	Eg	2015/9/13	-	-
2015/9/19	*D. sperconsus*	E	10-12-1	m	La	2015/9/16	2016/4/22	219
2015/9/19	*D. sperconsus*	F	11-3-3	f	La	2015/9/15	2016/4/24	222
2015/9/19	*D. sperconsus*	G	13-3-1	f	Eg	2015/9/17	2016/4/23	219
2015/9/19	*D. sperconsus*	H	7-15-2	m	La	2015/9/16	2016/4/24	221
2015/9/19	*D. sperconsus*	H	10-14-2	f	Eg	2015/9/19	2016/4/24	218
2015/9/19	*D. sperconsus*	H	20-3-2	m	La	2015/9/16	2016/4/22	219
2015/9/19	*D.* spp.	E	6-15-1	-	Eg	2015/9/17	-	-
2015/9/19	*D.* spp.	E	8-12-2	-	Eg	2015/9/17	-	-
2015/9/19	*D.* spp.	H	4-5-1	-	Eg	2015/9/17	-	-
2015/9/19	*D.* spp.	H	4-5-2	-	Eg	2015/9/19	-	-
2015/9/19	*D.* spp.	H	8-6-1	-	Eg	2015/9/17	-	-
2015/9/19	*D.* spp.	H	14-3-1	-	Eg	2015/9/17	-	-
2015/9/19	*D.* spp.	H	14-3-2	-	Eg	2015/9/17	-	-
2015/9/19	*D. sperconsus*	E	10-12-3	-	Eg	2015/9/17	-	-
2015/9/19	*D. sperconsus*	F	11-3-2	-	La	2015/9/14	-	-
2015/9/19	*D. sperconsus*	G	13-3-2	-	Eg	2015/9/18	-	-
2015/9/19	*D. sperconsus*	H	7-15-1	-	Eg	2015/9/17	-	-
2015/9/19	*D. sperconsus*	H	7-15-3	-	Eg	2015/9/17	-	-
2015/9/19	*D. sperconsus*	H	10-14-1	-	Eg	2015/9/17	-	-
2015/9/19	*D. sperconsus*	H	20-3-1	-	La	2015/9/15	-	-
2015/9/20	*D. sperconsus*	H	10-15-1	f	Eg	2015/9/19	2016/4/23	217
2015/9/22	*D.* spp.	E	8-12-1	-	Eg	2015/9/21	-	-
2015/9/22	*D.* spp.	G	13-8-1	-	Eg	2015/9/20	-	-
2015/9/22	*D.* spp.	H	10-12-1	-	Eg	2015/9/21	-	-
2015/9/28	*D. sperconsus*	E	7-5-2	f	La	2015/9/23	2016/4/25	215
2015/9/28	*D. sperconsus*	E	7-5-3	f	La	2015/9/24	2016/4/26	215
2015/9/28	*D. sperconsus*	E	7-5-4	m	La	2015/9/26	2016/4/24	211
2015/9/28	*D. sperconsus*	E	7-5-5	f	La	2015/9/25	2016/4/25	213
2015/9/28	*D. sperconsus*	E	8-11-2	m	La	2015/9/27	2016/4/22	208
2015/9/28	*D. sperconsus*	F	8-11-1	m	Co	2015/8/20	2016/4/22	246
2015/9/28	*D. sperconsus*	H	6-15-2	f	La	2015/9/24	2016/4/24	213
2015/9/28	*D. sperconsus*	H	6-15-3	m	La	2015/9/25	2016/4/22	210
2015/9/28	*D. sperconsus*	H	6-15-4	f	La	2015/9/24	2016/4/24	213
2015/9/28	*D.* spp.	E	6-12-1	-	Eg	2015/9/28	-	-
2015/9/28	*D.* spp.	G	13-6-2	-	Eg	2015/9/26	-	-
2015/9/28	*D.* spp.	H	13-11-1	-	Eg	2015/9/28	-	-
2015/9/28	*D. sperconsus*	E	7-5-1	-	La	2015/9/25	-	-
2015/9/28	*D. sperconsus*	E	7-5-6	-	Eg	2015/9/26	-	-
2015/9/28	*D. sperconsus*	E	8-11-3	-	Eg	2015/9/26	-	-
2015/9/28	*D. sperconsus*	H	6-15-5	-	Eg	2015/9/26	-	-
2015/9/28	*D. sperconsus*	H	6-15-6	-	Eg	2015/9/27	-	-
2015/10/5	*D.* spp.	E	6-9-2	-	Eg	2015/10/3	-	-
2015/10/19	*D. sperconsus*	E	16-5-1	m	Co	2015/8/20	2016/4/22	246

Summer generations: 22.8±1.6 (n=46)

Overwinter generation: 229.7±13.9 (n=37)

*The oviposition day was estimated as follows: when we first detected an egg in a cell on a certain day in the daily survey, we identified its oviposition day as the previous day; when we found a larva in a collected nest and recorded the spinning day of the larva, we estimated its oviposition day by counting back to include the total average time of the egg and larval periods from that day.

Supplementary Table S12. Rearing records of *Dipogon sperconsus* and *D.* spp. in 2016.

Nest-withdrawing date	Species	Site	No. of cell	Sex: f, female; m, male	Developmental stage at nest-withdrawing: Eg, egg; La, larva; Co, cocoon	Estimated oviposition day*	Emergence day	Duration from oviposition to emergence
2016/6/27	*D.* spp.	J	3-3-1	-	La	2016/6/24	-	-
2016/6/27	*D.* spp.	J	3-3-2	-	La	2016/6/25	-	-
2016/7/22	*D. sperconsus*	J	1-9-1	m	La	2016/7/21	2016/8/10	20
2016/7/22	*D. sperconsus*	J	1-9-2	m	La	2016/7/20	2016/8/15	26
2016/7/22	*D. sperconsus*	J	4-11-1	f	La	2016/7/18	2016/8/14	27
2016/7/22	*D. sperconsus*	J	4-11-2	f	La	2016/7/19	2016/8/11	23
2016/7/22	*D. sperconsus*	J	4-11-3		La	2016/7/18	2016/8/11	24
2016/7/22	*D. sperconsus*	J	1-9-3	-	La	2016/7/18	-	-
2016/7/22	*D. sperconsus*	J	4-11-5	-	Eg	2016/7/20	-	-
2016/7/22	*D. sperconsus*	J	4-11-6	-	Eg	2016/7/20	-	-
2016/7/22	*D. sperconsus*	J	4-11-7	-	Eg	2016/7/20	-	-
2016/7/23	*D. sperconsus*	J	4-11-1	-	Eg	2016/7/22	-	-
2016/7/24	*D. sperconsus*	J	4-11-1	m	Eg	2016/7/22	2016/8/11	20
2016/7/28	*D. sperconsus*	J	2-15-1	f	La	2016/7/23	2016/8/15	23
2016/7/28	*D. sperconsus*	J	2-15-2	m	La	2016/7/24	2016/8/16	23
2016/7/28	*D. sperconsus*	M	11-6-1	f	La	2016/7/25	2016/8/18	24
2016/7/28	*D. sperconsus*	M	11-6-2	m	Eg	2016/7/27	2016/8/19	23
2016/7/28	*D. sperconsus*	M	11-6-3	f	Eg	2016/7/27	2016/8/19	23
2016/7/28	*D.* spp.	M	10-3-2	-	Eg	2016/7/26	-	-
2016/7/28	*D. sperconsus*	M	11-6-4	-	Eg	2016/7/26	-	-
2016/8/5	*D.* spp.	J	2-2-1	-	Eg	2016/8/3	-	-
2016/8/12	*D. sperconsus*	J	5-5-1	f	Co	2016/9/11	2017/5/15	246
2016/8/12	*D. sperconsus*	J	5-5-2	m	Co	2016/9/10	2017/5/14	246
2016/8/12	*D. sperconsus*	J	5-9-1	m	La	2016/8/6	2017/5/13	280
2016/8/12	*D. sperconsus*	J	5-9-2	m	La	2016/8/6	2017/5/14	281
2016/8/12	*D. sperconsus*	J	5-9-3	f	La	2016/8/6	2017/5/15	282
2016/8/12	*D. sperconsus*	L	5-6-2	f	Eg	2016/8/10	2017/5/14	277
2016/8/12	*D.* spp.	J	4-20-1	-	La	2016/8/8	-	-
2016/8/12	*D. sperconsus*	L	5-6-1	-	Eg	2016/8/10	-	-
2016/8/17	*D. sperconsus*	K	11-6-1	f	La	2016/8/15	2017/5/15	273
2016/8/17	*D. sperconsus*	K	11-6-2	f	Eg	2016/8/15	2017/5/15	273
2016/8/17	*D. sperconsus*	M	3-9-1	f	Eg	2016/8/16	2017/5/15	272
2016/8/17	*D.* spp.	K	11-8-2	-	Eg	2016/8/15	-	-
2016/8/17	*D.* spp.	L	5-6-1	-	La	2016/8/13	-	-
2016/8/25	*D. sperconsus*	J	1-5-1	f	La	2016/8/20	2017/5/15	268
2016/8/25	*D. sperconsus*	J	2-15-1	m	La	2016/8/18	2017/5/13	268
2016/8/25	*D. sperconsus*	J	2-15-3	m	La	2016/8/21	2017/5/14	266
2016/8/25	*D. sperconsus*	J	3-3-1	f	La	2016/8/17	2017/5/14	270
2016/8/25	*D. sperconsus*	J	3-3-2	m	La	2016/8/18	2017/5/13	268
2016/8/25	*D. sperconsus*	J	5-11-1	m	Co	2016/9/9	2017/5/13	246
2016/8/25	*D. sperconsus*	K	11-6-2	m	La	2016/8/18	2017/5/14	269
2016/8/25	*D. sperconsus*	K	11-6-4	f	La	2016/8/20	2017/5/14	267
2016/8/25	*D. sperconsus*	L	8-5-1	f	Co	2016/9/9	2017/5/13	246
2016/8/25	*D. sperconsus*	L	8-5-2	f	La	2016/8/17	2017/5/15	271
2016/8/25	*D. sperconsus*	L	8-5-3	f	La	2016/8/18	2017/5/14	269
2016/8/25	*D. sperconsus*	L	8-5-4	f	La	2016/9/10	2017/5/14	246
2016/8/25	*D. sperconsus*	L	12-5-1	m	Co	2016/9/9	2017/5/13	246
2016/8/25	*D. sperconsus*	L	16-13-1	m	Co	2016/9/9	2017/5/13	246
2016/8/25	*D. sperconsus*	L	16-13-2	f	Co	2016/9/11	2017/5/15	246
2016/8/25	*D. sperconsus*	L	16-13-3	f	Co	2016/9/10	2017/5/14	246
2016/8/25	*D. sperconsus*	L	16-13-4	f	Co	2016/9/10	2017/5/14	246
2016/8/25	*D. sperconsus*	L	16-13-5	f	Co	2016/9/10	2017/5/14	246
2016/8/25	*D. sperconsus*	L	16-13-6	f	Co	2016/9/9	2017/5/13	246
2016/8/25	*D. sperconsus*	L	17-14-1	f	Co	2016/9/11	2017/5/15	246
2016/8/25	*D. sperconsus*	L	21-6-1	m	Co	2016/9/9	2017/5/13	246
2016/8/25	*D. sperconsus*	M	3-9-1	f	Co	2016/9/10	2017/5/14	246
2016/8/25	*D. sperconsus*	M	3-9-2	f	Co	2016/9/11	2017/5/15	246
2016/8/25	*D. sperconsus*	M	3-9-3	m	Co	2016/9/10	2017/5/14	246
2016/8/25	*D. sperconsus*	M	3-9-4	m	La	2016/8/18	2017/5/15	270
2016/8/25	*D.* spp.	J	2-2-1	-	La	2016/8/21	-	-
2016/8/25	*D. sperconsus*	J	1-5-2	-	Eg	2016/8/23	-	-
2016/8/25	*D. sperconsus*	J	1-5-3	-	Eg	2016/8/24	-	-
2016/8/25	*D. sperconsus*	M	3-9-5	-	La	2016/8/19	-	-
2016/8/26	*D.* spp.	J	2-15-1	-	Eg	2016/8/25	-	-
2016/9/1	*D. sperconsus*	J	1-3-2	f	La	2016/8/26	2017/5/15	262
2016/9/1	*D. sperconsus*	J	2-15-1	f	La	2016/8/26	2017/5/15	262
2016/9/1	*D. sperconsus*	J	2-15-2	m	La	2016/8/27	2017/5/12	258
2016/9/1	*D. sperconsus*	J	12-3-1	m	La	2016/8/25	2017/5/14	262
2016/9/1	*D. sperconsus*	K	1-1-1	m	La	2016/8/25	2017/5/14	262
2016/9/1	*D. sperconsus*	K	5-12-2	f	La	2016/8/27	2017/5/15	261
2016/9/1	*D. sperconsus*	L	2-13-2	f	La	2016/8/26	2017/5/14	261
2016/9/1	*D. sperconsus*	L	2-13-3	f	La	2016/8/27	2017/5/14	260
2016/9/1	*D. sperconsus*	M	5-12-1	m	Eg	2016/8/31	2017/5/14	256
2016/9/1	*D.* spp.	L	20-3-1	-	La	2016/8/25	-	-
2016/9/1	*D. sperconsus*	K	1-1-2	-	La	2016/8/27	-	-
2016/9/1	*D. sperconsus*	K	5-12-3	-	Eg	2016/8/30	-	-
2016/9/1	*D. sperconsus*	K	5-12-4	-	Eg	2016/8/30	-	-
2016/9/1	*D. sperconsus*	M	5-12-2	-	Eg	2016/8/30	-	-
2016/9/2	*D. sperconsus*	M	5-9-1	f	Eg	2016/9/1	2017/5/14	255
2016/9/2	*D. sperconsus*	J	1-9-1	f	Eg	2016/9/1	2017/5/14	255
2016/9/2	*D. sperconsus*	M	5-9-2	-	Eg	2016/9/1	-	-
2016/9/3	*D. sperconsus*	J	1-9-1	m	Eg	2016/9/2	2017/5/13	253
2016/9/3	*D.* spp.	K	5-8-1	-	Eg	2016/9/2	-	-
2016/9/7	*D. sperconsus*	J	2-15-1	f	La	2016/9/1	2017/5/15	256
2016/9/7	*D. sperconsus*	J	5-5-1	m	La	2016/9/1	2017/5/14	255
2016/9/7	*D. sperconsus*	J	5-5-2	f	La	2016/9/2	2017/5/14	254
2016/9/7	*D. sperconsus*	J	5-15-2	m	La	2016/9/3	2017/5/13	252
2016/9/7	*D. sperconsus*	J	5-15-3	f	La	2016/9/2	2017/5/15	255
2016/9/7	*D. sperconsus*	J	5-15-5	f	Eg	2016/9/5	2017/5/13	250
2016/9/7	*D. sperconsus*	J	15-15-1	m	La	2016/9/1	2017/5/13	254
2016/9/7	*D. sperconsus*	J	15-15-2	m	La	2016/9/2	2017/5/13	253
2016/9/7	*D. sperconsus*	J	15-15-3	f	La	2016/9/2	2017/5/15	255
2016/9/7	*D. sperconsus*	K	6-8-2	f	La	2016/9/1	2017/5/15	256
2016/9/7	*D. sperconsus*	K	6-8-3	f	La	2016/9/2	2017/5/15	255
2016/9/7	*D. sperconsus*	L	6-12-1	m	La	2016/9/1	2017/5/14	255
2016/9/7	*D. sperconsus*	L	8-15-1	f	La	2016/9/2	2017/5/14	254
2016/9/7	*D. sperconsus*	L	8-15-2	f	La	2016/9/3	2017/5/14	253
2016/9/7	*D. sperconsus*	L	9-3-1	m	Eg	2016/9/5	2017/5/15	252

2016/9/7	*D. sperconsus*	L	16-15-1	f	La	2016/9/2	2017/5/14	254
2016/9/7	*D. sperconsus*	L	18-11-1	f	Eg	2016/9/6	2017/5/15	251
2016/9/7	*D. sperconsus*	L	19-6-1	f	Eg	2016/9/5	2017/5/15	252
2016/9/7	*D. sperconsus*	L	19-6-2	m	Eg	2016/9/6	2017/5/14	250
2016/9/7	*D. sperconsus*	L	20-14-1	m	La	2016/9/2	2017/5/13	253
2016/9/7	*D. sperconsus*	L	20-14-2	m	La	2016/9/2	2017/5/13	253
2016/9/7	*D. sperconsus*	M	3-3-2	m	La	2016/9/5	2017/5/13	250
2016/9/7	*D. sperconsus*	M	5-5-2	f	La	2016/9/1	2017/5/15	256
2016/9/7	*D. sperconsus*	J	5-15-1	-	La	2016/9/3	-	-
2016/9/7	*D. sperconsus*	J	5-15-4	-	La	2016/9/4	-	-
2016/9/7	*D. sperconsus*	J	15-15-4	-	Eg	2016/9/5	-	-
2016/9/7	*D. sperconsus*	K	1-8-2	-	Eg	2016/9/5	-	-
2016/9/7	*D. sperconsus*	L	8-15-3	-	Eg	2016/9/6	-	-
2016/9/8	*D. sperconsus*	L	8-3-1	m	Eg	2016/9/7	2017/5/12	247
2016/9/8	*D. sperconsus*	L	9-3-1	f	Eg	2016/9/7	2017/5/15	250
2016/9/8	*D. sperconsus*	L	16-6-1	m	Eg	2016/9/7	2017/5/13	248
2016/9/8	*D.* spp.	L	18-3-1	-	Eg	2016/9/7	-	-
2016/9/16	*D. sperconsus*	J	1-14-1	m	Co	2016/9/9	2017/5/13	246
2016/9/16	*D. sperconsus*	J	4-15-1	f	La	2016/9/10	2017/5/14	246
2016/9/16	*D. sperconsus*	J	4-15-2	m	La	2016/9/11	2017/5/14	245
2016/9/16	*D. sperconsus*	J	5-15-1	m	La	2016/9/11	2017/5/13	244
2016/9/16	*D. sperconsus*	J	5-15-2	m	La	2016/9/10	2017/5/14	246
2016/9/16	*D. sperconsus*	J	12-15-1	m	La	2016/9/10	2017/5/12	244
2016/9/16	*D. sperconsus*	J	12-15-2	f	La	2016/9/13	2017/5/14	243
2016/9/16	*D. sperconsus*	J	12-15-3	f	La	2016/9/13	2017/5/14	243
2016/9/16	*D. sperconsus*	K	4-14-1	m	Eg	2016/9/14	2017/5/12	240
2016/9/16	*D. sperconsus*	K	15-15-1	m	La	2016/9/10	2017/5/15	247
2016/9/16	*D. sperconsus*	K	15-15-2	m	La	2016/9/13	2017/5/12	241
2016/9/16	*D. sperconsus*	L	6-12-1	f	La	2016/9/11	2017/5/15	246
2016/9/16	*D. sperconsus*	L	6-12-2	m	La	2016/9/12	2017/5/14	244
2016/9/16	*D. sperconsus*	L	18-3-1	m	La	2016/9/12	2017/5/14	244
2016/9/16	*D. sperconsus*	L	18-3-2	m	La	2016/9/10	2017/5/13	245
2016/9/16	*D. sperconsus*	L	18-3-4	f	La	2016/9/10	2017/5/15	247
2016/9/16	*D. sperconsus*	L	18-3-5	f	La	2016/9/12	2017/5/17	247
2016/9/16	*D. sperconsus*	L	18-3-6	f	Eg	2016/9/14	2017/5/17	245
2016/9/16	*D. sperconsus*	L	18-3-7	m	Eg	2016/9/14	2017/5/13	241
2016/9/16	*D. sperconsus*	L	18-3-8	f	Eg	2016/9/14	2017/5/15	243
2016/9/16	*D. sperconsus*	L	18-11-1	f	La	2016/9/9	2017/5/14	247
2016/9/16	*D. sperconsus*	L	18-11-3	f	La	2016/9/10	2017/5/14	246
2016/9/16	*D. sperconsus*	L	18-11-4	f	La	2016/9/10	2017/5/13	245
2016/9/16	*D. sperconsus*	L	19-9-2	f	La	2016/9/11	2017/5/15	246
2016/9/16	*D. sperconsus*	L	21-12-1	m	La	2016/9/10	2017/5/14	246
2016/9/16	*D. sperconsus*	L	21-12-2	m	La	2016/9/11	2017/5/15	246
2016/9/16	*D. sperconsus*	L	21-12-3	m	La	2016/9/10	2017/5/14	246
2016/9/16	*D. sperconsus*	L	21-12-5	f	La	2016/9/12	2017/5/14	244
2016/9/16	*D. sperconsus*	L	21-12-6	f	La	2016/9/12	2017/5/15	245
2016/9/16	*D. sperconsus*	L	21-12-7	f	La	2016/9/12	2017/5/13	243
2016/9/16	*D. sperconsus*	M	3-5-2	m	La	2016/9/10	2017/5/12	244
2016/9/16	*D. sperconsus*	M	3-5-3	f	La	2016/9/12	2017/5/13	243
2016/9/16	*D. sperconsus*	M	4-6-1	f	La	2016/9/10	2017/5/15	247
2016/9/16	*D. sperconsus*	M	5-2-1	f	La	2016/9/12	2017/5/14	244
2016/9/16	*D. sperconsus*	M	6-12-1	f	La	2016/9/8	2017/5/15	249
2016/9/16	*D. sperconsus*	M	14-2-1	f	La	2016/9/9	2017/5/15	248
2016/9/16	*D. sperconsus*	M	14-2-2	m	La	2016/9/10	2017/5/12	244
2016/9/16	*D. sperconsus*	M	14-2-3	f	La	2016/9/12	2017/5/15	245
2016/9/16	*D.* spp.	J	2-5-1	-	Eg	2016/9/14	-	-
2016/9/16	*D. sperconsus*	J	5-15-3	-	La	2016/9/13	-	-
2016/9/16	*D. sperconsus*	L	18-11-2	-	La	2016/9/11	-	-
2016/9/16	*D. sperconsus*	L	21-12-9	-	Eg	2016/9/14	-	-
2016/9/16	*D. sperconsus*	L	21-12-10	-	Eg	2016/9/15	-	-
2016/9/16	*D. sperconsus*	M	3-5-1	-	La	2016/9/11	-	-
2016/9/16	*D. sperconsus*	M	3-5-4	-	Eg	2016/9/14	-	-
2016/9/16	*D. sperconsus*	M	5-2-2	-	Eg	2016/9/14	-	-
2016/9/24	*D. sperconsus*	L	12-6-1	f	La	2016/9/18	2017/5/14	238
2016/9/24	*D. sperconsus*	M	9-3-1	m	La	2016/9/5	2017/5/9	246
2016/9/24	*D.* spp.	L	18-6-1	-	Eg	2016/9/22	-	-
2016/9/30	*D. sperconsus*	J	10-16-1	f	La	2016/9/24	2017/5/13	231
2016/9/30	*D. sperconsus*	J	10-16-2	f	La	2016/9/27	2017/5/13	228
2016/9/30	*D. sperconsus*	L	4-3-1	f	La	2016/9/24	2017/5/15	233
2016/9/30	*D. sperconsus*	L	4-3-2	m	La	2016/9/26	2017/5/13	229
2016/9/30	*D. sperconsus*	L	18-6-1	m	La	2016/9/25	2017/5/13	230
2016/9/30	*D. sperconsus*	M	2-12-1	m	Co	2016/9/10	2017/5/14	246
2016/9/30	*D. sperconsus*	M	2-12-6	m	Eg	2016/9/28	2017/5/14	228
2016/9/30	*D. sperconsus*	M	2-12-3	-	La	2016/9/26	-	-
2016/10/1	*D. sperconsus*	M	2-9-1	m	Eg	2016/9/30	2017/5/12	224
2016/10/5	*D. sperconsus*	K	12-15-1	f	Eg	2016/10/3	2017/5/14	223
2016/10/5	*D. sperconsus*	K	12-15-2	m	Eg	2016/10/3	2017/5/12	221
2016/10/5	*D. sperconsus*	K	12-15-3	f	Eg	2016/10/4	2017/5/15	223
2016/10/5	*D. sperconsus*	L	12-6-1	m	Co	2016/9/10	2017/5/14	246
2016/10/5	*D. sperconsus*	L	12-6-2	f	Co	2016/9/11	2017/5/15	246
2016/10/5	*D. sperconsus*	L	12-6-3	m	La	2016/9/28	2017/5/13	227
2016/10/5	*D. sperconsus*	L	20-12-1	m	Co	2016/9/9	2017/5/13	246
2016/10/5	*D.* spp.	L	4-6-2	-	Eg	2016/10/3	-	-
2016/10/5	*D.* spp.	M	2-9-3	-	Eg	2016/10/3	-	-
2016/10/5	*D. sperconsus*	K	12-15-4	-	Eg	2016/10/3	-	-
2016/10/6	*D.* spp.	M	2-15-1	-	Eg	2016/10/5	-	-

Summer generations: 23.3±2.1 (n=11)
Overwinter generation: 250.0±11.8 (n=127)

*The oviposition day was estimated as follows: when we first detected an egg in a cell on a certain day in the daily survey, we identified its oviposition day as the previous day; when we found a larva in a collected nest and recorded the spinning day of the larva, we estimated its oviposition day by counting back to include the total average time of the egg and larval periods from that day.

Supplementary Table S13. Newly emerging adults of *Dipogon speconsus* for 10 days of each month in 2013–2017 (see Figure 3a).

Year	10 days unit of month	No. emerging wasps			
		Females	Males	Total	Proportions of males
2013	Early June	0	0	0	-
	Mid June	0	0	0	-
	Late June	0	0	0	-
	Early July	2	0	2	0.00
	Mid July	2	1	3	0.33
	Late July	10	4	14	0.29
	Early Aug.	3	0	3	0.00
	Mid Aug.	4	4	8	0.50
	Late Aug.	4	5	9	0.56
	Early Sep.	0	0	0	-
	Mid Sep.	0	0	0	-
	Late Sep.	0	0	0	-
	Early Oct.	0	0	0	-
2014	Mid Apr.	0	0	0	-
	Late Apr.	0	0	0	-
	Early May	0	8	8	1.00
	Mid May	27	15	42	0.36
	Late May	0	0	0	-
	Early June	0	0	0	-
	Mid June	0	0	0	-
	Late June	0	0	0	-
	Early July	0	0	0	-
	Mid July	0	0	0	-
	Late July	6	5	11	0.45
	Early Aug.	1	4	5	0.80
	Mid Aug.	0	0	0	-
	Late Aug.	1	0	1	0.00
	Early Sep.	0	0	0	-
	Mid Sep.	0	0	0	-
	Late Sep.	0	0	0	-
	Early Oct.	0	0	0	-
2015	Mid Apr.	0	0	0	-
	Late Apr.	13	7	20	0.35
	Early May	1	0	1	0.00
	Mid May	0	0	0	-
	Late May	0	0	0	-
	Early June	0	0	0	-
	Mid June	0	0	0	-
	Late June	0	0	0	-
	Early July	0	0	0	-
	Mid July	0	0	0	-
	Late July	5	3	8	0.38
	Early Aug.	0	0	0	-
	Mid Aug.	5	3	8	0.38
	Late Aug.	20	10	30	0.33
	Early Sep.	0	0	0	-
	Mid Sep.	0	0	0	-
	Late Sep.	0	0	0	-
	Early Oct.	0	0	0	-
2016	Mid Apr.	0	1	1	1.00
	Late Apr.	20	16	36	0.44
	Early May	0	0	0	-
	Mid May	0	0	0	-
	Late May	0	0	0	-
	Early June	0	0	0	-
	Mid June	0	0	0	-
	Late June	0	0	0	-
	Early July	0	0	0	-
	Mid July	0	0	0	-
	Late July	0	0	0	-
	Early Aug.	0	1	1	1.00
	Mid Aug.	5	4	10	0.44
	Late Aug.	0	0	0	-
	Early Sep.	0	0	0	-
	Mid Sep.	0	0	0	-
	Late Sep.	0	0	0	-
	Early Oct.	0	0	0	-
2017	Mid Apr.	0	0	0	-
	Late Apr.	0	0	0	-
	Early May	0	1	1	1.00
	Mid May	70	56	126	0.44
	Late May	0	0	0	-

Supplementary Table S14. Eggs laid by female wasps of *Dipogon superconsus* and eggs that would develop into non-overwinterng and overwintering individuals for 10 days of each month in 2013–2016 (see Figure 3b).

Year	10 days unit of month	Estimated no. of eggs laid					Estimated no. of eggs that would develop into:			
		Females	Males	Unknown	Total	Proportions of males	Non-overwintering wasps	Overwintering wasps	Total	Proportions of overwintering larvae
2013	Early June	0	0	0	0	-	0	0	0	-
	Mid June	4	0	2	6	0.00	4	0	4	0.00
	Late June	3	2	1	6	0.40	5	0	5	0.00
	Early July	10	3	0	13	0.23	13	0	13	0.00
	Mid July	0	0	0	0	-	0	0	0	-
	Late July	4	5	0	9	0.56	8	0	8	0.00
	Early Aug.	9	12	3	24	0.57	9	12	21	0.57
	Mid Aug.	1	1	0	2	0.50	0	2	2	1.00
	Late Aug.	5	3	0	8	0.38	0	8	8	1.00
	Early Sep.	4	9	0	13	0.69	0	13	13	1.00
	Mid Sep.	12	3	7	22	0.20	0	15	15	1.00
	Late Sep.	0	0	0	0	-	0	0	0	-
	Early Oct.	0	0	0	0	-	0	0	0	-
2014	Mid Apr.	0	0	0	0	-	0	0	0	-
	Late Apr.	0	0	0	0	-	0	0	0	-
	Early May	0	0	0	0	-	0	0	0	-
	Mid May	0	0	0	0	-	0	0	0	-
	Late May	0	0	0	0	-	0	0	0	-
	Early June	0	0	0	0	-	0	0	0	-
	Mid June	0	0	0	0	-	0	0	0	-
	Late June	0	0	0	0	-	0	0	0	-
	Early July	5	5	5	15	0.50	10	0	10	0.00
	Mid July	2	4	3	9	0.67	6	0	6	0.00
	Late July	0	0	2	2	-	0	0	0	-
	Early Aug.	1	0	0	1	0.00	1	0	1	0.00
	Mid Aug.	0	1	0	1	1.00	0	1	1	1.00
	Late Aug.	3	2	0	5	0.40	0	5	5	1.00
	Early Sep.	5	2	1	8	0.29	0	7	7	1.00
	Mid Sep.	4	2	3	9	0.33	0	6	6	1.00
	Late Sep.	2	0	1	3	0.00	0	2	2	1.00
	Early Oct.	0	0	0	0	-	0	0	0	-
2015	Mid Apr.	0	0	0	0	-	0	0	0	-
	Late Apr.	0	0	0	0	-	0	0	0	-
	Early May	0	0	0	0	-	0	0	0	-
	Mid May	0	0	0	0	-	0	0	0	-
	Late May	0	0	0	0	-	0	0	0	-
	Early June	0	0	0	0	-	0	0	0	-
	Mid June	0	0	0	0	-	0	0	0	-
	Late June	2	2	0	4	0.50	4	0	4	0.00
	Early July	3	1	1	5	0.25	4	0	4	0.00
	Mid July	0	0	0	0	-	0	0	0	-
	Late July	22	9	12	43	0.29	31	0	31	0.00
	Early Aug.	3	4	6	13	0.57	7	0	7	0.00
	Mid Aug.	1	6	0	7	0.86	0	7	7	1.00
	Late Aug.	5	1	0	6	0.17	0	6	6	1.00
	Early Sep.	4	4	0	8	0.50	0	8	8	1.00
	Mid Sep.	5	3	4	12	0.38	0	8	8	1.00
	Late Sep.	5	3	5	13	0.38	0	8	8	1.00
	Early Oct.	0	0	0	0	-	0	0	0	-
2016	Mid Apr.	0	0	0	0	-	0	0	0	-
	Late Apr.	0	0	0	0	-	0	0	0	-
	Early May	0	0	0	0	-	0	0	0	-
	Mid May	0	0	0	0	-	0	0	0	-
	Late May	0	0	0	0	-	0	0	0	-
	Early June	0	0	0	0	-	0	0	0	-
	Mid June	0	0	0	0	-	0	0	0	-
	Late June	0	0	0	0	-	0	0	0	-
	Early July	0	0	0	0	-	0	0	0	-
	Mid July	2	1	3	6	0.33	3	0	3	0.00
	Late July	3	4	1	8	0.57	7	0	7	0.00
	Early Aug.	2	2		4	0.50	0	4	4	1.00
	Mid Aug.	8	4	1	13	0.33	0	12	12	1.00
	Late Aug.	5	5	4	14	0.50	0	10	10	1.00
	Early Sep.	31	32	5	68	0.51	0	63	63	1.00
	Mid Sep.	19	8	4	31	0.30	0	27	27	1.00
	Late Sep.	3	5	1	9	0.63	0	8	8	1.00
	Early Oct.	2	1	0	3	0.33	0	3	3	1.00

Supplementary Table S15. The 50% overwintering-egg days and average temperatures for 7 day-, 10 day- and 14 day-periods immediately before Aug. 1, when the earliest one of the 50% overwintering-egg day was observed in 2013-2017. Correlaltion coefficiencies between the 50% overwintering-egg days and the average temperatures for 7 day-, 10 day- and 14 day-period are 0.69, 0.96 and 0.99, respectively. Note that Figure 3a illustrates the cases of 10 day- and 14-day period data.

Year	The 50% overwintering-egg day	The 50% overwintering-egg day counted from July 1(days)	Average of mean temperature for:		
			7 days from July 26 to Aug.1 (°C)	10 days from July 23 to Aug. 1 (°C)	14 days from July 19 to Aug. 1 (°C)
2013	Aug. 3	33	26.33	26.52	26.10
2014	Aug. 6	36	26.67	27.13	26.59
2015	Aug. 7	37	27.44	26.99	26.64
2016	Aug. 1	31	26.61	26.11	25.75

Supplementary Table S16. Daily mean temperatures and its averages for 7 day-, 10 day-, and 14 day-period immediately before the target days during June 1 and Aug. 15 in 2013-2016, and correlation coefficiencies between the 50% overwintering-egg days and the average temperatures of 7 day- (A1), 10 day- (A2), and 14 day-periods (A3). Note that Figure 4b illustrates daily changes of correlation coefficiencies with the average temperatures of 10 day- and 14 day-periods (A2 and A3). Red-colored numbers show the same ones as in Supplementary Table S15.

Calendar day	Mean temperature of the day *i*				Average of mean temperature for: (A1) 7 day-period just before the day *i* (°C)				(A2) 10 day-period just before the day *i* (°C)				(A3) 14 day-period just before the day *i* (°C)				Correlation coefficient (r) between the day of 50% overwintering egg-day and:		
	2013	2014	2015	2016	2013	2014	2015	2016	2013	2014	2015	2016	2013	2014	2015	2016	A1	A2	A3
June 1	18.70	24.00	21.60	18.80															
2	20.30	22.70	22.80	16.80															
3	21.20	21.30	20.40	18.10															
4	21.10	20.90	18.20	19.10															
5	21.80	20.20	14.00	18.30															
6	21.80	21.30	16.50	20.90															
7	20.90	21.20	18.80	19.20	20.83	21.66	18.90	18.74									0.25		
8	20.90	22.70	19.50	22.00	21.14	21.47	18.60	19.20									-0.01		
9	20.70	22.90	20.10	21.30	21.20	21.50	18.21	19.84									-0.26		
10	22.00	22.90	21.20	22.80	21.31	21.73	18.33	20.51	20.94	22.01	19.31	19.73					-0.39	0.13	
11	22.80	21.50	20.00	23.90	21.56	21.81	18.59	21.20	21.35	21.76	19.15	20.24					-0.53	-0.18	
12	25.80	20.30	22.70	22.70	22.13	21.83	19.83	21.83	21.90	21.52	19.14	20.83					-0.67	-0.47	
13	26.90	18.50	23.10	21.00	22.86	21.43	20.77	21.84	22.47	21.24	19.41	21.12					-0.68	-0.59	
14	26.60	20.70	21.80	23.20	23.67	21.36	21.20	22.41	23.02	21.22	19.77	21.53	22.25	21.51	20.05	20.58	-0.72	-0.66	-0.24
15	22.80	22.40	22.80	23.80	23.94	21.31	21.67	22.67	23.12	21.44	20.65	22.08	22.54	21.39	20.14	20.94	-0.69	-0.74	-0.41
16	24.30	22.10	21.60	21.60	24.46	21.20	21.89	22.71	23.37	21.52	21.16	22.15	22.83	21.35	20.05	21.28	-0.61	-0.67	-0.55
17	24.50	21.20	22.80	23.00	24.81	20.96	22.11	22.74	23.73	21.52	21.56	22.53	23.06	21.34	20.22	21.63	-0.56	-0.69	-0.64
18	24.90	19.80	20.40	23.50	25.11	20.71	22.17	22.69	24.13	21.23	21.65	22.68	23.34	21.26	20.38	21.94	-0.52	-0.67	-0.70
19	23.40	21.50	18.70	20.10	24.77	20.89	21.60	22.31	24.40	21.09	21.51	22.56	23.45	21.36	20.71	22.07	-0.54	-0.64	-0.69
20	19.60	22.90	21.40	24.20	23.73	21.51	21.36	22.77	24.16	21.09	21.53	22.70	23.29	21.47	21.06	22.31	-0.79	-0.69	-0.75
21	18.80	22.80	21.80	24.40	22.61	21.81	21.36	22.94	23.76	21.22	21.71	22.75	23.14	21.59	21.28	22.68	-0.99	-0.73	-0.87
22	20.70	20.70	21.80	23.20	22.31	21.57	21.21	22.86	23.25	21.26	21.62	22.80	23.13	21.44	21.44	22.76	-1.00	-0.83	-0.88
23	21.70	21.20	23.30	22.80	21.94	21.44	21.46	23.03	22.73	21.53	21.64	22.98	23.20	21.32	21.67	22.87	-0.93	-0.96	-0.85
24	22.10	21.70	23.40	21.20	21.60	21.51	21.54	22.77	22.28	21.63	21.80	22.78	23.21	21.24	21.83	22.76	-0.82	-0.95	-0.79
25	23.50	23.10	24.60	22.30	21.40	21.99	22.14	22.60	22.35	21.70	21.98	22.63	23.26	21.35	22.16	22.64	-0.20	-0.91	-0.66
26	20.60	23.40	21.70	22.00	21.00	22.26	22.57	22.87	21.98	21.83	21.99	22.67	22.89	21.57	22.09	22.59	0.06	-0.80	-0.74
27	22.70	23.00	22.00	21.80	21.44	22.27	22.66	22.53	21.80	22.01	21.91	22.55	22.59	21.89	22.01	22.65	0.28	-0.64	-0.94
28	22.50	22.60	18.70	20.90	21.97	22.24	22.21	22.03	21.56	22.29	21.74	22.29	22.29	22.03	21.79	22.49	0.86	-0.19	-0.98
29	23.60	22.30	21.40	22.10	22.39	22.47	22.16	21.87	21.58	22.37	22.01	22.49	22.35	22.02	21.69	22.36	0.54	-0.12	-0.92
30	23.20	22.50	20.80	23.80	22.60	22.66	21.80	22.01	21.94	22.33	21.95	22.45	22.27	22.05	21.63	22.52	-0.06	-0.44	-0.95
July 1	23.80	23.80	22.20	25.30	22.84	22.96	21.63	22.60	22.44	22.43	21.99	22.54	22.22	22.24	21.59	22.69	-0.47	-0.78	-0.86
2	23.30	23.80	22.60	26.80	22.81	23.06	21.34	23.24	22.70	22.74	22.07	22.90	22.11	22.52	21.74	22.92	-0.70	-0.77	-0.69
3	23.10	20.80	23.20	27.80	23.17	22.69	21.56	24.07	22.84	22.70	22.06	23.40	22.09	22.47	22.06	23.47	-0.95	-0.93	-0.71
4	23.80	23.30	20.80	26.90	23.33	22.73	21.39	24.80	23.01	22.86	21.80	23.97	22.39	22.50	22.02	23.66	-0.96	-0.91	-0.84
5	26.10	22.00	21.30	27.90	23.84	22.64	21.76	25.80	23.27	22.75	21.47	24.53	22.91	22.44	21.99	23.91	-0.98	-0.95	-0.97
6	26.40	22.80	20.70	27.00	24.24	22.71	21.66	26.50	23.85	22.69	21.37	25.03	23.31	22.59	21.91	24.19	-0.99	-0.98	-0.99
7	27.20	23.40	21.60	27.20	24.81	22.84	21.77	26.99	24.30	22.73	21.33	25.57	23.71	22.75	21.79	24.50	-0.99	-0.99	-0.98
8	28.20	26.10	24.10	24.20	25.44	23.17	22.04	26.83	24.87	23.08	21.87	25.90	24.14	23.06	21.84	24.71	-1.00	-0.99	-0.96
9	27.30	26.60	25.20	23.40	26.01	23.57	22.41	26.34	25.24	23.51	22.25	26.03	24.41	23.31	21.88	24.79	-0.97	-0.98	-0.94
10	27.50	24.50	26.40	26.20	26.64	24.10	22.87	26.11	25.67	23.71	22.81	26.27	24.91	23.39	22.21	25.09	-0.90	-0.99	-0.95
11	27.60	25.50	26.20	27.10	27.19	24.41	23.64	26.14	26.05	23.88	23.21	26.45	25.26	23.57	22.51	25.47	-0.84	-0.98	-0.96
12	27.50	25.20	27.30	26.50	27.39	24.87	24.50	25.94	26.47	24.02	23.68	26.42	25.61	23.76	23.13	25.87	-0.72	-0.95	-0.97
13	26.30	24.00	27.90	25.90	27.37	25.04	25.53	25.79	26.79	24.34	24.15	26.23	25.81	23.88	23.59	26.14	-0.48	-0.89	-0.98
14	24.50	25.50	27.80	25.40	26.99	25.34	26.41	25.53	26.86	24.56	24.85	26.08	25.90	24.09	24.09	26.26	0.05	-0.79	-0.97
15	23.50	25.70	27.00	22.90	26.31	25.29	26.83	25.34	26.60	24.93	25.42	25.58	25.88	24.23	24.44	26.09	0.44	-0.49	-0.95
16	25.80	27.30	26.20	24.60	26.10	25.39	26.97	25.51	26.54	25.38	25.97	25.34	26.06	24.48	24.69	25.93	0.51	0.06	-0.90
17	26.00	27.40	22.80	26.20	25.89	25.80	26.46	25.51	26.42	25.78	26.09	25.24	26.26	24.95	24.66	25.81	0.81	0.44	-0.85
18	26.40	26.30	23.40	25.80	25.71	25.91	26.06	25.33	26.24	25.80	26.02	25.40	26.45	25.16	24.85	25.74	0.97	0.46	-0.75
19	23.90	23.90	25.30	25.20	25.20	25.73	25.77	25.14	25.90	25.53	26.03	25.58	26.29	25.30	25.14	25.54	0.97	0.37	-0.59
20	24.60	24.80	27.10	24.70	24.96	25.84	25.66	24.97	25.61	25.56	26.10	25.43	26.16	25.44	25.59	25.38	0.90	0.76	-0.09
21	26.20	25.60	26.30	24.40	25.20	25.86	25.44	24.83	25.47	25.57	26.11	25.16	26.09	25.60	25.93	25.18	0.85	0.90	0.50
22	25.50	26.60	24.40	25.10	25.49	25.99	25.07	25.14	25.27	25.71	25.82	25.02	25.90	25.64	25.95	25.24	0.25	1.00	0.68
23	26.80	27.00	23.90	25.80	25.63	25.94	24.74	25.31	25.32	26.01	25.42	25.01	25.86	25.66	25.86	25.41	-0.20	0.72	0.62
24	26.80	28.20	26.40	24.80	25.74	26.06	25.26	25.11	25.55	26.28	25.28	24.95	25.81	25.93	25.86	25.31	0.35	0.53	0.84
25	27.30	29.40	27.50	24.20	25.87	26.50	25.84	24.89	25.93	26.65	25.33	25.08	25.79	26.21	25.95	25.11	0.77	0.41	0.87
26	27.20	28.80	26.80	24.30	26.34	27.20	26.06	24.76	26.07	26.80	25.39	25.05	25.77	26.46	25.91	24.95	0.70	0.43	0.83
27	26.80	25.50	26.90	26.10	26.66	27.30	26.03	24.96	26.15	26.61	25.80	25.04	25.81	26.57	25.84	24.96	0.60	0.63	0.77
28	25.80	23.90	26.40	27.30	26.60	27.06	26.04	25.37	26.09	26.37	26.10	25.19	25.90	26.46	25.74	25.10	0.54	0.81	0.68
29	24.70	26.00	27.00	27.00	26.49	26.97	26.41	25.64	26.17	26.58	26.27	25.37	25.99	26.48	25.74	25.39	0.75	0.84	0.56
30	27.40	27.30	27.60	27.10	26.57	27.01	26.94	25.83	26.45	26.83	26.32	25.61	26.10	26.48	25.84	25.57	0.94	0.72	0.52
31	25.90	27.90	28.60	27.10	26.44	26.97	27.26	26.16	26.42	27.06	26.55	25.88	26.09	26.51	26.26	25.64	0.99	0.81	0.88
Aug. 1	26.50	27.30	28.80	27.40	**26.33**	**26.67**	**27.44**	**26.61**	**26.52**	**27.13**	**26.99**	**26.11**	**26.10**	**26.59**	**26.64**	**25.75**	**0.69**	**0.96**	**0.99**
2	26.10	24.90	28.80	26.70	26.17	26.11	27.73	26.96	26.45	26.92	27.48	26.20	26.26	26.66	26.89	25.86	0.26	0.96	1.00
3	25.30	24.70	28.50	26.60	25.96	26.00	27.96	27.03	26.30	26.57	27.69	26.38	26.31	26.65	26.99	25.99	0.27	0.76	0.98
4	25.90	26.20	28.70	27.70	25.97	26.33	28.29	27.09	26.16	26.25	27.81	26.73	26.29	26.69	27.16	26.23	0.40	0.45	0.93
5	26.90	27.50	29.10	28.20	26.29	26.54	28.59	27.26	26.13	26.12	28.04	27.12	26.39	26.76	27.50	26.45	0.42	0.27	0.83
6	27.30	27.50	27.50	27.30	26.27	26.57	28.57	27.29	26.18	26.32	28.10	27.24	26.42	26.79	27.76	26.56	0.42	0.28	0.78
7	26.30	27.10	28.50	27.90	26.33	26.46	28.56	27.40	26.23	26.64	28.31	27.30	26.39	26.71	27.91	26.78	0.34	0.39	0.64
8	27.80	24.40	27.40	27.60	26.51	26.04	28.36	27.43	26.54	26.48	28.35	27.36	26.42	26.36	27.90	27.02	0.15	0.31	0.34
9	28.80	22.90	27.30	27.00	26.90	25.76	28.14	27.47	26.68	26.04	28.32	27.35	26.54	25.94	27.94	27.21	-0.05	0.15	0.08
10	29.00	25.00	27.50	25.50	27.43	25.80	28.00	27.31	26.99	25.75	28.21	27.19	26.69	25.90	27.98	27.17	-0.12	0.06	0.08
11	29.10	25.30	26.50	25.30	27.89	25.67	27.69	26.97	27.25	25.55	27.98	26.98	26.93	26.00	27.99	27.03	-0.18	-0.01	0.14
12	28.50	24.60	26.30	26.70	28.11	25.26	27.29	26.76	27.49	25.52	27.73	26.98	27.20	25.90	27.94	27.01	-0.29	-0.14	0.05
13	26.90	25.70	25.20	27.60	28.06	25.00	26.96	26.80	27.65	25.62	27.40	27.08	27.16	25.79	27.76	27.04	-0.41	-0.32	-0.05
14	27.70	26.30	25.80	27.20	28.26	24.89	26.57	26.70	27.83	25.63	27.11	27.03	27.29	25.67	27.56	27.05	-0.48	-0.43	-0.19
15	28.40	26.60	26.00	27.50	28.34	25.20	26.37	26.69	27.98	25.54	26.80	26.96	27.43	25.62	27.36	27.06	-0.52	-0.51	-0.30

Supplementary Table S17. The results of the consecutive daily investigations in a total of 68 nests. E, egg of *Dipogon sperconsus*; e, egg of brood parasitic *D. nagasei* (Note that this species exceptionally lays 1–5 eggs in a cell); N, spider without wasp egg; /, cell partition; –, no nesting.

Nest No.	Nesting start day or period	New installation date	1st day	2nd day	3rd day	4th day	5th day	6th day	7th day	8th day
**2014** (Investigation period: 0508–1028)										
B-7-15	0707	0707	3cells (E/E/E)	–	–					
B-7-12	0707–0714	0714	2cells (E/E)	2cells (E/E)	–	–				
A-6-9	0714	0714	–	–	1cell (N)	–	–			
A-7-9	0714	0714	–	–	1cell (5e)	–	–			
B-7-2	0714	0714	–	–	–	1cell (E)	–	–		
B-7-15	0714	0714	–	–	–	–	2cells (e/e)	–	–	
B-6-8	0728	0728	2cells (E/N)	–	–					
B-6-6	0728	0728	–	1cell (5e)	–	–				
B-6-3	0728	0728	–	–	2cells (3e/4e)	–	–			
A-14-12	0728–0811	0811	1cell (E)	–	2cells (E/N)	–	–			
A-8-12	0811–0825	0825	1cell (N)	1cell (E)	–	–				
A-12-12	0811–0825	0825	1cell (E)	–	–					
A-9-12	0903	0903	1cell (E)	–	–					
A-10-15	0903–0908	0908	1cell (E)	–	–					
**2015** (Investigation period: 0605–1028)										
B-11-15	0711	0711	–	1cell (E)	–	–				
B-15-15	0711	0711	–	–	1cell (E)	–	–			
A-13-3	0725	0725	–	–	1cell (E)	–	–			
B-11-13	0725	0725	–	–	1cell (E)	–	–			
B-12-4	0725	0725	–	–	2cells (E/E)	–	–			
C-11-3	0725	0725	–	–	2cells (E/E)	–	–			
C-13-14	0725	0725	–	–	2cells (E/N)	–	–			
A-6-6	0725	0725	–	–	–	1cell (E)	–	–		
A-15-6	0725	0725	–	–	–	3cells (E/E/E)	–	–		
A-16-12	0725	0725	–	–	–	3cells (E/N/N)	–	–		
D-2-3	0725	0725	–	–	–	2cells (E/N)	–	–		
A-6-2	0725	0725	–	–	–	–	3cells (E/E/N)	–	–	
A-15-7	0725	0725	–	–	–	–	1cell (N)	–	–	
C-10-6	0725	0725	–	–	–	–	1cell (N)	–	–	
C-11-3	0725	0725	–	–	–	–	2cells (E/N)	–	–	
D-2-15	0725	0725	–	–	–	–	1cell (N)	–	–	
A-10-3	0725	0725	–	–	–	–	–	4 cells (E/E/N/E)	–	–
A-11-12	0725	0725	–	–	–	–	–	1cell (E)	–	–
B-14-15	0725	0725	–	–	–	–	–	3cells (3e/N/E)	–	–
D-2-12	0725	0725	–	–	–	–	–	2cells (E/N)	–	–
A-6-12	0725	0725	–	–	–	–	–	–	2cells (N/E)	–
A-9-15	0725	0725	–	–	–	–	–	–	1cell (E)	–
A-10-2	0725	0725	–	–	–	–	–	–	2cells (E/E)	–
C-13-12	0725	0725	–	–	–	–	–	–	3cells (N/2e/E)	–
D-2-12	0725	0725	–	–	–	–	–	–	2cells (E/N)	–
A-6-6	0725	0725	–	–	–	–	–	–	–	4 cells (N/E/5e/4e)
C-10-15	0725	0725	–	–	–	–	–	–	–	2cells (E/E)
A-6-9	0803	0803	2cells (E/E)	–	–					
A-8-9	0803	0803	2cells (E/E)	–	–					
C-5-6	0803	0803	2cells (E/E)	–	–					
C-10-8	0803	0803	3cells (E/E/E)	–	–					
A-6-5	0803	0803	–	2cells (E/E)	–	–				
A-8-3	0803	0803	–	2cells (E/E)	–	–				
B-6-6	0803	0803	–	1cell (E)	–	–				
A-8-9	0811	0811	1cell (N)	–	–					
D-2-15	0905	0905	1cell (E)	–	–					
D-6-15	0905	0905	1cell (E)	–	–					
A-14-9	0912	0912	2cells (E/N)	–	–					
D-2-9	0912	0912	2cells (E/N)	1cell (E)	–	–				
D-8-12	0905–0912	0912	1cell(E)	1cell (E)	–	–				
D-14-6	0912	0912	–	1cell (E)	–	–				
D-14-12	0912	0912	2cells (E/E)	–	–					
D-10-15	0919	0919	1cell (E)	–	–					
**2016** (Investigation period: 0604–1021)										
A-4-11	0715–0722	0722	1cell (E)	1cell (E)	–	–				
A-2-15	0817–0825	0825	1cell (E)	–	–					
D-5-9	0901	0901	2cells (E/E)	–	–					
A-1-9	0901	0901	1cell (E)	1cell (E)	–	–				
B-5-8	0901	0901	–	2cells (E/N)	–	–				
C-8-3	0907	0907	2cells (E/N)	–	–					
C-9-3	0901–0907	0907	1cells (E)	–	–					
C-16-6	0907	0907	1cell (E)	–	–					
C-18-3	0907	0907	1cell (E)	–	–					
D-2-9	0930	0930	1cell (E)	–	–					
D-2-15	1005	1005	1cell (E)	–	–					

End of supple.
